# Can trusted authorities change minds on anti-LGBTQ norms: Evidence from an experiment in Ghana

**DOI:** 10.1371/journal.pone.0304698

**Published:** 2024-08-22

**Authors:** Michael Yekple, Zlatin Mitkov

**Affiliations:** 1 School of Politics, Security and International Affairs, University of Central Florida, Orlando, Florida, United States of America; 2 Department of Political Science, School of Public and International Affairs, North Carolina State University, Raleigh, North Carolina, United States of America; NYU Grossman School of Medicine: New York University School of Medicine, UNITED STATES

## Abstract

Across numerous African societies, a prevalent resistance to LGBTQ rights is evident. While prominent strides have been made on LGBTQ rights in various parts of the world, the African context has witnessed limited progress. Public opinion polls indicate that influential figures have succeeded in altering public sentiment towards LGBTQ rights in Western countries, yet such progress remains elusive in the African context. Mechanisms effective in shifting public attitudes toward embracing LGBTQ rights remain largely unexplored, especially in the African context. In this paper, we consider whether endorsement messages conveyed by trusted authorities possess the potential to foster a shift in attitudes towards embracing LGBTQ rights among a nationally representative sample of respondents in Ghana. Through a factorial experiment, we find that there are varying impacts of messaging from distinct trusted authorities in shaping attitudes toward embracing LGBTQ rights. Notably, pro-LGBTQ messaging from traditional and co-partisan leaders yields modest effects, while messaging from religious leaders exhibits no discernible impact. These findings hold significance for determining strategic focal points in potential endeavors to advance LGBTQ rights within the African context.

## Introduction

Can trusted authorities change minds on anti- lesbian, gay, bisexual, transgender, and queer (LGBTQ) norms in conservative African societies? Though a strong movement has enhanced respect for LGBTQ people globally, same-sex expressions and gender-nonconforming relationships have been prohibited or criminalized in most parts of Africa. For instance, out of 69 countries with national laws criminalizing same-sex relations between consenting adults globally, 33 of these are based in Africa [1]. While same-sex relationships and politics surrounding these remain contentious in many parts of the world, the extent to which anti-LGBTQ norms shift in developing countries over time and the factors that make these possible are not well known and deserve proper scholarly attention.

The lack of LGBTQ norm acceptance in Africa is a complex issue inspired by various historical, cultural, religious, and political factors. Political homophobia in Africa has deep-rooted causes, with two prominent factors being the perception of homosexuality as a colonial imposition of foreign ideals and the argument that it pervades African values. Many Africans view homosexuality through a historical lens, associating it with the legacy of colonialism. As a result, some Africans perceive homosexuality as a foreign concept that threatens their indigenous traditions and customs. This perception has led to a strong resistance to accepting homosexuality as a legitimate expression of human sexuality, often viewed as an affront to African identity and thus un-African 35 [2, 3]. But such claims are disputed as ahistorical as homosexual practices and homosexuals have always existed in African cultures [4–6]. Furthermore, scholars like M’Baye have observed that in countries like Uganda and Senegal, politicians have exploited the issue of homosexuality as a means to divert attention from pressing economic problems that they have failed to address adequately [2]. By stoking public sentiment against LGBTQ rights, politicians can rally their base and deploy homophobia as a political tool [7]. This tactic effectively shifts blame and avoids accountability for their governance failures. The use of anti-homosexual rhetoric as a political diversion not only perpetuates discrimination but also hampers progress on critical economic and social fronts. It is a strategy that has regrettably been used in various African countries, exacerbating the challenges faced by LGBTQ individuals while avoiding addressing more significant issues affecting their societies [2].

In recent years, issues surrounding LGBTQ rights have taken center stage in public debate in various parts of Africa and have generated heated debate and controversy, with opposition to sexual minority rights and fear of or anxiety about people whose sexuality or gender identity does not conform to a narrow band of heterosexual norms [8].

Africa’s LGBTQ inclusion landscape is diverse due to varying attitudes, laws, and acceptance. While some countries decriminalized same-sex relationships (e.g., South Africa, Cape Verde, Lesotho, Rwanda, Mali, and Mozambique), others retain harsh laws (e.g., Chad, Sudan, Nigeria, Ghana, Egypt, Tunisia, Tanzania, Uganda, and Mauritania, Nigeria). There has been recent progress and hope for advancement of tolerance when Angola and Botswana decriminalized same-sex relationships in 2019. However, Kenya upheld laws criminalizing homosexual relationships in that same year. Over 30 African countries still outlaw consensual same-sex relationships [9].

Attempts by Western governments, including the United States, to punish African countries for enacting anti-LGBTQ laws have sometimes backfired, risking further animosity and violence from the public [10]. In many African countries, there is evidence of internal debate and disagreements among Africans on the subject of homosexuality, with many citizens holding divergent views on the matter [11].

A common conclusion anyone can reach is that the systematic discrimination and criminalization of LGBTQ norms in Africa is counter to human rights conventions, public health best practices, and sound economic development. Hostility towards homosexuality in Africa also goes against global human rights conventions and the prevailing consensus on sound economic development [8]. LGBTQ individuals in Africa face increased vulnerabilities due to exclusion from opportunities and services.

While there is significant discussion in the policy and scholarly debates on various aspects of LGBTQ issues in Africa, how to change attitudes and improve LGBTQ rights has been mainly ignored. At the regional level, Izugbara et al. (2020) [12] explored regional legal instruments that could be leveraged to advance the rights of LGBTQ persons in the region [13]. Their examination revealed seven significant treaties formulated between 1981 and 2018 by the African Union that could serve this purpose. The authors recognized the binding agreements emphasize AU member-states’ duty to protect all citizens, regardless of sexual orientation. The authors noted that while they promote inclusive agendas, explicitly mentioning sexual and reproductive health rights, equality, and freedom, they lack direct reference to LGBTQ individuals and mechanisms for member-state accountability. They argue that strengthening commitment to these instruments can improve legal frameworks and advance LGBTQ inclusion and well-being in the region [13].

The endeavor to shift attitudes towards tolerance of LGBTQ rights in Africa presents a demanding undertaking. Considering how entrenched anti-LGBTQ norms are in Africa, changing people’s attitudes against sexual minorities will require the adoption of progressive legislation and policies. However, the policy choices in these conservative societies have potential electoral ramifications for politicians who pursue them. Pushing for progressive policies to normalize LGBTQ values can put politicians who pursue these agendas in a clash with the public’s moral norms, thus endangering political prospects. This has the potential of constraining the most progressive politicians, who may take into account the political expediency of not offending their electoral constituencies.

There remains a noticeable lack of research exploring effective strategies geared toward shifting attitudes and fostering the embrace of LGBTQ rights. This gap underscores the need for scholarship that extends beyond merely dissecting the barriers of LGBTQ rights and, instead, delving into strategies for catalyzing transformative change. One of the mechanisms in the literature for improving public tolerance and modifying opinion on sensitive issues is the role of trusted authorities [13, 14]. Trusted authorities such as religious and traditional leaders constitute important figures in African societies. Studies have highlighted their ability to change people’s willingness to accept certain policy issues as accepting former insurgents into the community [13]. However, we do not know if such authority figures can change minds on LGBTQ issues. Our research seeks to contribute to this area by interrogating the role of trusted authorities in changing minds toward embracing LGBTQ rights.

Through an experiment with a nationally representative sample conducted in Ghana, we observed a nuanced role of trusted authorities in influencing attitude change toward LGBTQ rights. Different authority figures appeal to distinct values to foster tolerance: religious leaders emphasize compassion and benevolence, traditional authorities highlight communal harmony and coexistence, and political leaders convey messages of tolerance. Our findings demonstrate that while pro-LGBTQ messages from traditional leaders yield modest attitude shifts towards embracing LGBTQ rights, similar messaging from religious and political authorities lacks such impact. These results underscore the importance of identifying strategic focal points for potential efforts to advance LGBTQ rights within the African context.

## Theoretical motivation

Previous research shows that trusted authorities can change public attitudes and shift the interpretation of social norms [13, 14]. Insights drawn from political science and psychology scholarship posit that cues from trusted authorities, moral role models, and influential societal members possess the capacity to cause shifts in both individual dispositions and prevailing societal norms by disseminating affirmative messages around specific issues. It is a prevalent phenomenon for individuals to shape their beliefs and choices guided by cues emanating from their trusted figures [15–18]. Additionally, the proclamations and actions of these leaders serve as standards for societal benchmarks, as individuals frequently assume that their fellow community constituents will observe and be swayed by the messages and actions of these leaders. This process consequently engenders shifts in personal attitudes and behaviors [19–21].

Recent research centered on the role of messages conveyed by trusted societal figures to shape attitudes across various matters has produced mixed outcomes. For instance, a recent study in Nigeria by Blair et al. (2021) reveals that influential figures wield significant potential in altering attitudes toward the reintegration of former extremists and adherents of the Boko Haram terrorist group back into society. This is particularly relevant in scenarios where former combatants and members of such groups have encountered ostracism and resistance upon attempting to reintegrate into the social fabric. Conversely, a recent study in Burkina Faso found the limitation of impact carried by messages from religious leaders in reshaping attitudes toward violent extremism [22]. These juxtaposed findings naturally raise inquiries concerning the viability of such interventions in fostering acceptance and tolerance for LGBTQ individuals.

Generally, the landscape of LGBTQ rights and acceptance has undergone significant transformation in recent decades, partly due to the influence of well-known non-LGBTQ public figures who have played instrumental roles in reshaping societal views. Often wielding substantial cultural, political, or artistic influence, these figures have leveraged their platforms to advocate for inclusivity, challenge prejudices, and champion equal rights for LGBTQ individuals. Through their words, actions, and visible support, these figures have catalyzed critical conversations that have helped reshape societal norms and foster greater acceptance. In the United States, A Pew Research poll has found that 66 percent of Americans polled say well-known non-LGBTQ public figures were instrumental in helping society to be more accepting of LGBTQ persons [22]. Prominent figures like former U.S. President Barack Obama, whose presidency marked a pivotal moment in advancing LGBTQ rights, played vital roles in changing attitudes. The influence of non-LGBTQ allies like Emma Watson, a globally recognized actress and UN Women Goodwill Ambassador, demonstrates the importance of allyship in advancing LGBTQ acceptance. Watson has consistently used her platform to advocate for gender equality and LGBTQ rights, emphasizing the interconnectedness of various social justice issues. She has demonstrated how allies can play a crucial role in driving a more inclusive society by amplifying marginalized voices and advocating for change across intersectional lines.

Thus, prominent public figures have undertaken endeavors to shift attitudes toward the acceptance of LGBTQ individuals and the endorsement of their rights over the past few decades, achieving substantial success in many Western contexts [22]. Nonetheless, such efforts have yielded comparatively modest results in the developing world, especially in the African context (see S1 Fig) [23, 24]. There exists a dearth of extensive research probing the efficacy of messages in reshaping attitudes and norms regarding LGBTQ issues. This motivates us to study how trusted authorities can change social norms and pave the way for social change on LGBTQ rights.

Studying the role of trusted authorities on their potential to change attitudes toward embracing LGBTQ rights further allows us to study how the public balances deeply held anti-LGBTQ norms and political affiliations if the two conflict. Does the public alter its political preferences against that of political leaders if they do not uphold the public’s rigid moral norms? How the public balances deeply held anti-LGBTQ norms and the nature of political affiliations give political leaders open to making progress on LBGTQ rights or not supporting anti-LGBTQ legislation useful information to manage political risk-taking behaviors. The study contributes to scholarship on African political behavior, which is generally understudied or restricted to a limited scope of issues that influence the public’s electoral choices.

## Research context

Our research context is Ghana, where widespread anti-LGBTQ norms have solidified as dominant moral values within Ghanaian society. Recent research findings indicate that a staggering 93 percent of Ghanaians vehemently oppose LGBTQ rights [24], with a majority advocating for the state to criminalize both their activities and advocacy efforts.

In recent years, Ghana has become mired in a contentious debate surrounding LGBTQ rights. A group of the country’s legislature members has introduced a uniquely punitive legislation before the floor of parliament called the "Human Sexual Rights and Ghanaian Family Values" bill, and it has been under discussion since August 2021 [25]. This proposed legislation seeks to criminalize same-sex relationships, transgender identities, and advocacy for LGBTQ rights. It is worth noting that most Members of Parliament support this bill publicly. Politicians likely cannot oppose the bill publicly because of negative political repercussions as the bill has a large amount of public support.

The implications of this legislation have ignited outrage and apprehension among human rights activists. If enacted and assented to by the executive arm of government, it could lead to severe penalties, including sentences of up to 10 years in prison for LGBTQ individuals and those who support their rights, express sympathy, or provide social and medical assistance [25].

In July 2023, Ghana’s Supreme Court dismissed a legal challenge to prevent parliament from enacting the stringent anti-LGBT bill [26]. This ruling clears the path for the bill’s approval. The LGBTQ community in Ghana is deeply concerned about the potential hazards they may encounter if this legislation becomes law. There is a palpable fear that it could incite violence and persecution against sexual and gender minorities.

This proposed legislation has garnered widespread condemnation from human rights advocates and international observers, who argue that it signifies a substantial regression in LGBTQ rights within the country [27].

We argue that trusted authorities potentially hold the promise of effecting progress in LGBTQ rights within the present Ghanaian context. The prospect of this outcome is underscored by earlier research demonstrating the capacity of trusted authorities, for instance, traditional leaders, to influence shifts in societal norms [28–30]. Trusted authorities serve as crucial nodes for shaping and altering public opinions [30, 31]. In Ghana’s political and cultural fabric, trusted authorities manifest as religious leaders, traditional figures, and political leaders. We posit that these trusted figures can leverage distinct values: compassion and benevolence, communal harmony and coexistence, and tolerance. Given their pivotal roles in molding public sentiment, we explore how these trusted authorities in Ghana can alter public attitudes on the contentious issue of LGBTQ rights, a matter of intense social and cultural debate.

### The source of influence of trusted authorities in Ghana

Traditional and religious leaders as trusted authorities are influential in Ghanaian society. Ghana has a robust chieftaincy system where traditional leaders wield substantial influence and command deep respect within their communities. Traditional leaders draw their authority and power from rich historical and cultural roots and ancestry [31]. Traditional leaders have been governing their communities since pre-colonial eras and continue to receive recognition from the modern state, with their roles acknowledged as pivotal across various facets of Ghanaian society [32]. The significance of traditional leaders is evident in their integration into Ghana’s constitutional framework post-independence, where they coexist with formal governance structures. Under the 1992 Constitution of Ghana, the authority of chiefs and traditional councils is explicitly guaranteed and upheld under Article 270 [33]. Despite rapid modernization, traditional chiefs maintain a pivotal role, particularly in land tenure, local governance, and cultural heritage preservation. They are esteemed as custodians of the land, managing it for the benefit of present and future generations, with constitutional safeguards in place to uphold the integrity of their position, including a prohibition on active participation in partisan politics. Consequently, traditional leaders and chiefs in Ghana retain a distinct and influential role in various aspects of societal life, including land management, local governance, and the preservation of cultural heritage and traditions [34, 35]. Stemming from their historical influence and power, traditional leaders continue to have significant influence and respect in Ghanaian society, as a recent Afrobarometer survey showed that 55 percent of Ghanaians trust traditional leaders [36].

Religious leaders are equally influential in Ghana. With the arrival of Christianity and Islam during colonial and pre-colonial periods, religious dynamics in Ghana underwent significant transformation. Missionaries and Islamic scholars established churches and mosques, respectively, spreading their faiths across the region. These religious institutions became centers of community life, providing spiritual guidance, education, healthcare, and social support. Religious leaders have since emerged as influential figures in Ghanaian society. According to an Afrobarometer study, religious leaders are among the trusted authority figures in Ghana, with 64 percent of the population having trust in them, thus bestowing on them an enormous influence on public discourse [36]. The post-independence era saw the consolidation of religious leaders’ influence as Ghana navigated political and social changes. Religious institutions have remained an essential aspect of Ghanaian society amidst an evolving political landscape, continually serving as moral compasses and advocating for social justice, human rights, and political accountability [29]. Their moral authority transcended religious boundaries, earning them respect across diverse segments of society.

In contemporary Ghana, the importance of religious leaders persists in various spheres of life. Politically, they play active roles, endorsing candidates and influencing electoral outcomes. Their endorsements carry significant weight, especially among religiously inclined voters [36]. Religious leaders frequently engage in socio-economic issues, advocating for poverty alleviation, healthcare access, and education reform. Their pronouncements on these matters resonate with policymakers and the public alike, shaping discourse and policy agendas [37, 38].

The importance and influence of religious leaders in Ghana are thus deeply rooted in historical traditions and contemporary realities. From traditional spiritualists to modern-day preachers and imams, they serve as guardians of morality, advocates for social change, and mediators between tradition and modernity. Their enduring influence underscores the pivotal role of religion in shaping Ghanaian identity and society. Religious leaders will undoubtedly remain influential sources of public opinion.

### The use of political homophobia in Ghana

For political leaders in Ghana, especially, their status as trusted authorities carry electoral ramifications. Within the specific political landscapes of Ghana, characterized by deep polarization and strong alignments with the two major political parties, efforts by political leaders to reshape public attitudes regarding anti-LGBTQ norms could potentially constrict public political affiliations and party loyalty. Political leaders desiring to effectuate changes in LGBTQ norms might hesitate if uncertain whether such shifts in public sentiment would alter their political allegiances in a landscape marked by polarization and intense competition.

Due these dynamics, the strategic utilization of homophobia within the political landscape has proven to be an effective tool for garnering public support in recent times. Politicians employ this tactic by depicting homosexuality as a foreign, morally corrupting influence on Ghanaian culture. It is common for politicians to use homophobia as a diversionary tactic employed by politicians in post-independence African states, deflecting attention away from their inability to address substantive economic and political challenges faced by the population [11].

However, the portrayal of homosexuality as a foreign intrusion into African culture in Africa is not historically accurate. Scholars agree that homosexuality and homosexual practices have a longstanding and nuanced history deeply embedded in various African cultures. Numerous historical accounts and anthropological studies substantiate the existence of same-sex relationships and practices across the continent long before the arrival of external influences [4, 5]. These relationships often held unique cultural significance, reflecting the complexity and diversity inherent in African societies [39].

The effective utilization of political homophobia within the African political landscape is connected to the influence of religion. Across the continent, religious beliefs exert a significant impact on societal attitudes towards homosexuality. Religious doctrines cast homosexuality as incompatible with moral and cultural values, reinforcing negative perceptions within communities [11]. This has created a fertile ground for the strategic deployment of homophobia as a political tool, as political actors exploit these sentiments, framing their opposition to homosexuality as a defense of religious and cultural integrity.

Ghanaian political elites have exploited the tendency of LQBTQ rights to ruffle religious sensitivities in a country where the majority are religious. Being a highly religious country with 71 percent Christian and 20 percent Muslim, religious leaders in Ghana have projected a strong anti-LGBTQ stance since the issues became salient in public debates over the past decade, with Christian and Muslim groups, including Ghana Catholic Bishops Conference (GCBC), the Presbyterian Church of Ghana (PCG) and the Coalition of Muslim Organizations, Ghana (COMOG) throwing their support behind the bill seeking the criminalization of LGBTQ rights in the country [40, 41].

Political elites in opposition or in power have trafficked in populist anti-LGBTQ rhetoric, knowing its popularity among a receptive religious audience. The former president John Atta Mills, upon returning from an African Union Summit where the then UN Secretary-General Ban-Ki Moon gave a speech deploring African governments’ poor record on LGBTQ rights, countered those claims by insisting that LGBTQ rights are against African values and culture [42]. A Sitting speaker of Ghana’s parliament in 2017 declared to a group of religious leaders that parliament would not bow to foreign pressure to legalize gay rights in Ghana, adding that LGBTQ rights are antithetical to Ghana’s culture and values [42]. Recently, politicians across the political spectrum have exploited anti-LGBTQ rhetoric in supporting an anti-LGBTQ bill. The main sponsor of the anti-LGBTQ bill, who is also a member of parliament, without any evidence, accused his political opponent of being sponsored by foreign LGBTQ lobby groups, clearly seeking to exploit the issue in an electoral cycle [43]. The same member of parliament had previously claimed Western countries and interest groups targeted him to unseat him for his stance on LGBTQ issues [43].

Parliamentary debates on the bill were used to draw public disaffection towards members who had reservations about aspects of the bill, casting insinuations on them as LGBTQ persons. As a result, no member of parliament could vote against the bill, possibly for fear of public disapproval. The bill was unanimously approved by all members across the political spectrum.

Opponents of the sitting president have persistently called upon him to disclose his stance while exerting pressure to secure his assent to the bill [44]. Opponents of the sitting president are well aware that refusing to assent to the bill will create political ill-will among the public. The president thus faces the delicate task of navigating public sentiment and political dynamics surrounding the contentious legislation and is thus incentivized to avoid losing public support. In the upcoming election, the former president and current opposition presidential candidate has affirmed to religious leaders that the Christian faith to which he belongs does not endorse LGBTQ rights, aligning himself with the prevailing anti-LGBTQ sentiments within the religious community [45]. This move by the former president is believed to have been calculated to avoid losing support among the religious community.

## Research design

The study’s data is based on a survey experiment with a nationally representative sample of 821 Ghanaians citizens residing in Ghana. Field work started on the 24th of April 2023 and ended on 15th May 2023.The study has IRB approval and was designed as a cross-section and representative nationwide sample survey undertaken in Ghana. Ghana has 16 administrative regions and 261 districts. We combined probability and non-probability sampling techniques to ensure equal distribution of urban and rural districts where the respondents were selected. First, all 16 administrative regions were represented in the sample selection. A stratified multistage random sampling technique was then adopted to select the respondents from the regions. For the purposes of stratification, the region was stratified into urban and rural districts, from which two districts were selected. The district of the regional capitals was purposely selected to represent urban areas, but the district of the rural areas was randomly selected using random numbers.

Ethics Statement and Procedures. Written and verbal consent was obtained by each of the participants by the enumerators. Our main priority in the study was to ensure that our enumerators follow the required safety and ethical standards and because of that the few procedures were followed. First, enumerators provided and read consent forms in both written and verbal form to the participants and explained the procedure of the experiment, including the nature of the questions. We worked closely with local researchers to navigate the local communities in the most safe and discrete manner. Second, this study employed deception as the messages we used were not in fact made by the respective authority figures. After the experiment, the enumerators debriefed the participants and explained to them in verbal and written form that the messages were not real and that we deployed deception to make the treatments appear realistic. To the best of our knowledge the study did not generate threats to our participants, enumerators, and supervisors. This study was IRB approved by a team led by the coordinator of the administration. See S1 and S2 Files for a more detailed overview of the selection procedures, the recruitment process, the demographics of the collected set of respondents, and the dataset.

Turning to the survey instrument itself in the first section, respondents were asked to indicate their demographic details, religious and political leanings, and level of interest in politics. The study employs a factorial design, ensuring that the respondents were randomly assigned to one of the four treatment groups (see S2 Fig). Treatment one consisted of a placebo scenario in which respondents are informed about the importance of living a healthy life. Treatment two presented respondents with a pro-LGTBQ endorsement message by a traditional leader aiming to increase public support for the rights of these communities. In comparison, in treatment three, a political leader from the respondent’s party has expressed support for the pro-LGBTQ policies. On the other hand, respondents in treatment four received a pro-LGBTQ endorsement message from a local religious figure. For Muslim respondents, the endorsement message was attributed to the national Chief Imam, whom all Ghanaian Muslims revere and take spiritual guidance from. Whereas for Christians, the messages were attributed to a leading Christian leader. For political leaders, the endorsement message was attributed to the respondent’s member of parliament or a leading member of the respondent’s political party, depending on which is more realistic. For traditional leaders, the messages were attributed to the most prominent traditional leader in the region in which the respondents reside.

Following the treatment, respondents were asked three types of dependent variables about the LGBTQ community in Ghana using an 11-point scale. The first question: *"Will you support or oppose LGBTQ rights in Ghana*? *"*, aimed to capture their level of support for the idea of providing rights to the LGBTQ community and individuals. The second question is part of the first set of outcomes: "*How likely is it that you are going to vote for political candidates who are pro-LGBTQ*?" was used to capture participants’ interest in voting for pro-LGBTQ candidates in the country. Questions one and two were aimed at the political side of public life. The third question measured the economic dimensions by asking respondents’ willingness to do business and live close by to members of the LGBTQ community. The final questions targeted the socio-cultural aspect of the issues. Specifically, the question *"I understand the difficulties that members of the LGBTQ community in Ghana are experiencing"*) measured respondents’ level of support for LGBTQ rights in the country and their perceived understanding of the problems that these communities are facing. Similarly, respondents were asked to indicate their willingness to allow *"Members of the LGBTQ ……*.. *to participate in community meetings*.*"* which provides a somewhat different measure of the public’s willingness to allow such individuals and groups to participate in community meetings, thus being directly in contact with them. Hence, we posit that this set of post-treatment questions allowed us to capture the expected effects of the endorsement cues on participants’ pro-LGBTQ inclinations across political, cultural, and economic issues.

We anticipated some potential challenges to our research design stemming from the political and social context surrounding LGBTQ issues in Ghana. These challenges potentially render our research treatments less plausible in the minds of our respondents. It is conceivable that our respondents harbored doubts regarding messages attributed to trusted authorities, particularly those authority figures frequently featured in the media discussing national matters. Notably, political authorities, including members of parliament, who often weigh in on national issues, may have endorsement messages attributed to them on LGBTQ issues doubted. Respondents may assume that, given the prominent public profile of these officials and their proclivity for commenting on critical national matters, any statements related to LGBTQ issues would have been widely covered in the media. Consequently, we suspect that respondents might perceive our treatments as implausible, believing that had their respective political leaders made such statements concerning LGBTQ issues, they would have encountered these statements through media channels.

Conversely, we have reason to believe that treatments from other trusted authorities, particularly those not renowned for their public pronouncements on national controversies, may not face the same degree of doubt. To illustrate, we anticipate that treatments emanating from traditional leaders, who infrequently involve themselves in national controversies, would not be as readily perceived as implausible when compared to those originating from political leaders.

We have conceived two potential strategies to mitigate this bias. The first involves soliciting actual trusted authorities to provide recorded messages in their voices, explicitly endorsing support for LGBTQ rights, which we would then present to our respondents. However, we have found this approach to be fraught with challenges. This is primarily due to the possible adverse repercussions it could entail for the respective trusted authorities, especially political figures who may face political and electoral backlash from specific demographics that strongly oppose LGBTQ rights. Consequently, these trusted authorities may consider it prohibitively costly to lend their voices to public endorsements of LGBTQ rights, even if they privately support such stances.

The second approach involves addressing this potential bias by leveraging respondents’ political awareness, specifically assessing the extent to which they closely follow national politics. This measure allows us to gauge their likelihood of perceiving the endorsements from trusted authorities as less believable. We postulate that respondents who closely monitor national politics are better equipped to make informed guesses about whether the respective trusted authorities have indeed issued endorsements in support of LGBTQ rights. Consequently, they are more likely to find the treatment less believable.

To execute this, we introduce an interaction between respondents’ level of political interest and each of the four endorsement treatments in our models. These interaction terms enable us to detect any potential divergent effects among respondents who are more politically informed compared to those who are less informed. The underlying expectation is that respondents with a higher level of political awareness may exhibit greater skepticism toward the plausibility of pro-LGBTQ endorsement cues provided by local religious, political, or traditional leaders. If, however, no significant differences emerge based on respondents’ level of political interest, we can reasonably conclude that the believability of the scenarios among our respondents is within an acceptable range.

### Inclusivity in global research

Additional information regarding the ethical, cultural, and scientific considerations specific to inclusivity in global research is included in the S1 File.

## Results

In Tables 1–3 and S3–S5 Figs, we present the outcomes of our experiment, employing proportion tests and logistic regression to analyze the effects of different treatments while controlling for age, gender, region of residence, ethnicity, partisanship, and level of political interest. These are established techniques for assessing and contrasting various treatment effects in experiments with factorial design. Notably, the data reveals a substantial level of opposition to LGBTQ rights, with over 85 percent of respondents expressing such sentiments. Examining Table 1 and S3 Fig, a significant disparity emerges between respondents exposed to the placebo health story (Placebo Cue) and those exposed to the endorsement message from their local traditional leader (Traditional cue) regarding support for LGBTQ rights. Only 2 percent of those exposed to the placebo expressed willingness to support such rights, compared to 9.5 percent among those who received the traditional leader’s endorsement. This difference of approximately 7.4 percent holds statistical significance, confirming the influence of cues from traditional leaders in Ghana–as expected in Hypothesis (H1). Additionally, respondents exposed to the placebo health story are, on average, 6.3 percent less likely to endorse LGBTQ rights compared to their counterparts who receive an endorsement message from a co-partisan political leader. This effect, which aligns with Hypothesis H1d, is statistically significant. Nevertheless, from a policy standpoint, as in the first comparison, the framing effect falls short in its ability to substantially alter the prevailing negative sentiment surrounding LGBTQ rights. Further analysis, involving a comparison between respondents’ willingness to support LGBTQ rights in the context of the placebo health story (2%) and the endorsement cue from a relevant religious leader (3.4%), does not yield statistically significant differences. This trend persists when contrasting the traditional leader’s endorsement cue (9.5%) with endorsement treatments from political (6.3%) or religious leaders (3.4%). Overall, the study’s results underscore the influence of cues from traditional leaders on attitudes toward LGBTQ rights, as indicated by a statistically significant albeit modest shift in support. However, other cues from political and religious leaders do not have significant effects.

**Table 1:** Ghanaians willingness to support LGBTQ rights.

Treatment Type/Cue	Treatment Type/Cue	Framing Effect
Placebo—Health Story	Endorsement by a Traditional Leader	7.4 percent***
2 percent	9.5 percent	
Placebo—Health Story	Endorsement by a Political Leader	4 percent**
2 percent	6.3 percent	
Placebo Health Story	Endorsement by a Religious Leader	1.3 percent
2 percent	3.4 percent	
Endorsement by a Traditional Leader	Endorsement by a Political Leader	3 percent
9.5 percent	6.3 percent	
Endorsement by a Traditional Leader	Endorsement by a Religious Leader	6 percent**
9.5 percent	3.4 percent	
Endorsement by a Political Leader	Endorsement by a Religious Leader	3 percent
6.3 percent	3.4 percent	

*Test of Proportions*. *p < 0.1

**p < 0.05

***p < 0.01; The comparisons are made horizontally. The first two columns of the table outline the type of cues and column one is compared to column two thus outlining the treatment effects. The dependent variable is binary in which one (1) stands for support for LGBTQ rights and zero (0) stands for lack of support for the rights of these communities. Higher percentages indicate a higher proportion of respondents’ support rights for the LGTBQ community.

**Table 2:** Ghanaians willingness to vote for political candidates supporting pro-LGBTQ policies.

Treatment Type/Cue	Treatment Type/Cue	Framing Effect
Placebo Health Story	Endorsement by a Traditional Leader	0.004 percent
7 percent	7.4 percent	
Placebo Health Story	Endorsement by a Political Leader	2 percent
7 percent	9 percent	
Placebo Health Story	Endorsement by a Religious Leader	3 percent
7 percent	4 percent	
Endorsement by a Traditional Leader	Endorsement by a Political Leader	0.005 percent
7.3 percent	8 percent	
Endorsement by a Traditional Leader	Endorsement by a Religious Leader	3.4 percent
7.3 percent	4 percent	
Endorsement by a Political Leader	Endorsement by a Religious Leader	3.6 percent
9 percent	5 percent	

*Test of Proportions*. *p < 0.1, **p < 0.05, ***p < 0.01; The comparisons are made horizontally. The first two columns of the table outline the type of cues and column one is compared to column two thus outlining the treatment effects. The dependent variable is binary in which one (1) indicates the willingness to vote for political candidates supporting pro-LGBTQ policies and zero (0) indicate the lack of such willingness to vote for pro-LGBTQ candidates. Higher percentages indicate a higher proportion of respondents are willing to support political candidates with pro-LGTBQ policies.

**Table 3:** Ghanaians willingness to do business with members of the LGBTQ communities.

Treatment Type/Cue	Treatment Type/Cue	Framing Effect
Placebo Health Story	Endorsement by a Traditional Leader	0.002 percent
12 percent	12.5 percent	
Placebo Health Story	Endorsement by a Political Leader	1.5 percent
12 percent	11 percent	
Placebo Health Story	Endorsement by a Religious Leader	4 percent
12 percent	8.4 percent	
Endorsement by a Traditional Leader	Endorsement by a Political Leader	1.7 percent
12.5 percent	11 percent	
Endorsement by a Traditional Leader	Endorsement by a Religious Leader	4 percent
12.5 percent	8.4 percent	
Endorsement by a Political Leader	Endorsement by a Religious Leader	2.3 percent
11 percent	8.4 percent	

*Test of Proportions*. *p < 0.1, **p < 0.05, ***p < 0.01; The comparisons are made horizontally. The first two columns of the table outline the type of cues and column one is compared to column two thus outlining the treatment effects. The dependent variable is binary in which one (1) indicates respondent’s willingness to do business with LGBTQ rights and zero (0) indicates the lack of support willingness among respondents to do business with the LGBTQ communities. Higher percentages indicate a higher proportion of respondents are willing to do business with the LGBTQ communities.

Proceeding to Table 2 and S4 Fig, which outline treatment effects on respondents’ willingness to vote for pro-LGBTQ candidates, the results demonstrate that support for such candidates ranges from 2 percent to 9.5 percent, while opposition to them hovers around 80 percent. Notably, endorsement cues from the various trusted authorities fail to significantly alter the level of support for pro-LGBTQ candidates. Therefore, hypothesis six (H6) and its variations are unsupported as the endorsement cues from local traditional, political, or religious figures fail to elicit increased support for pro-LGBTQ political candidates among our respondents.

Turning to Table 3 and S5 Fig, examining respondents’ willingness to engage in business with LGBTQ community members, we again find that endorsement cues do not yield robust effects across different scenarios. Whether endorsed by local traditional leaders or political figures, these cues only manage to sway around 12 percent of respondents towards such business engagements with the LGBTQ community. Consequently, hypothesis three (H3) is not supported because, across all treatments and the placebo group, statistically significant differences in willingness to do business with the LGBTQ community are absent, with a general opposition above 80 percent.

Next, we interact the treatment conditions with respondents’ level of political interest (See Tables 17–36 in S1 File). The objective is to assess whether our treatment interventions might have been biased by the potential skepticism among respondents regarding the authenticity of endorsement messages. This skepticism could have arisen when individuals with high political interest and engagement contemplated whether their traditional, co-religious, or co-partisan leaders had indeed made such declarations, as reported in the media. S6 Fig illustrates the interaction between respondents’ level of interest and the traditional cue. The results do not indicate a relationship between respondents’ levels of political interests, the endorsement cues from their local traditional leaders and their levels of support for the idea of providing rights to the LGBTQ community compared to the placebo group. S7 Fig shows the interaction term between respondents’ level of political interest and their willingness to support pro-LGBTQ candidates. However, again, the results do not indicate any systematic difference between individuals with different levels of political interest and their support for pro-LGBTQ candidates. Similarly, in S8 Fig, we interact with respondents’ willingness to do business with members of the LGBTQ community based on the former’s level of political interest. The results do not show a systematic pattern that individuals with higher levels of political interest have higher or lower self-reported willingness to do business with LGBTQ members and communities.

Consequently, it can be said that interacting respondents’ level of political interest with each of the treatment conditions does not lead to robust and systematic changes in the treatment effects compared to the baseline models. Despite our concern that respondents with higher levels of political interest might be less willing to believe the authenticity of the pro-LGBTQ endorsement cues, thus affecting their willingness to support pro-LGBTQ policies and political figures, we do not see such effects in the results. The findings of this study offer some evidence supporting the capacity of endorsement cues to foster pro-LGBTQ sentiments compared to the placebo condition. The results remain practically the same even after conducting the analysis only with those respondents who have passed the post-treatment manipulation check which amounts to 83%. While this result is promising, it should be interpreted while keeping in mind that our sample still exhibits a notably high level of intolerance towards the LGBTQ community, exceeding 80 percent.

## Discussion

In this section, we delve into the implications stemming from the notably inconclusive outcomes of the endorsement cues across various contexts. Initially, it is crucial to acknowledge that endorsement messages conveyed by traditional leaders and co-partisan political figures yield a discernible level of support, with treatment effects ranging from 4 to 7 percent support for LGBTQ rights. Nevertheless, the prevailing negative sentiment pervasive among Ghanaians, reaching an overwhelming 85 percent in opposition to granting rights to the LGBTQ community, diminishes the impact of the cues, despite their statistical significance. It is imperative to recognize that the efficacy of endorsement cues from religious, traditional, or co-partisan political leaders may be constrained by several factors.

First, the underpinning factor lies within the framework of a deeply ingrained, broad-based resistance to LGBTQ rights in Ghanaian society. This societal disposition potentially engenders conflict between one’s staunch anti-LGBTQ norms and the cues conveyed by trusted authorities. In essence, anti-LGBTQ priors wield substantial influence, ultimately eclipsing the priming effects of endorsements from trusted authorities. Regardless, endorsements emanating from traditional leaders exhibit a comparatively more potent ability to shift perspectives regarding anti-LGBTQ norms when juxtaposed with cues from co-partisan political leaders and religious leaders of one’s faith.

Additionally, the persuasive influence of one’s co-partisan political leaders surpasses that of religious leaders representing the respondents’ faith. This nuanced divergence in effects can be delineated through insights garnered from the extensive cues’ literature. A plausible explanation emerges, attributing these differential effects to the varying degrees of trust reposed in the respective trusted authorities by the respondents. Should we adopt this line of reasoning in the literature, a resounding deduction surfaces–traditional leaders stand as considerably more trustworthy and revered figures among our respondents. This attestation holds merit, as it aligns with the pattern of differential effects discernible in response to endorsements from different authoritative sources.

These complex dynamics prompt us to ponder the intricate terrain of tolerance and the broader spectrum of homophobia. It is crucial to acknowledge that the journey from endorsing the decriminalization of LGBTQ rights to embracing non-discrimination against LGBTQ individuals in one’s social interactions is marked by a substantial chasm. This chasm is reflective of the interplay between societal norms, personal biases, and deeply ingrained perceptions.

In essence, while endorsement cues can indeed spark shifts in respondents’ stances toward endorsing LGBTQ rights, the enduring reticence exhibited in accommodating LGBTQ individuals within their immediate social contexts is a testament to the multifaceted nature of tolerance and the complex facets of homophobia. This divergence between endorsing rights and translating that endorsement into tangible, inclusive interpersonal dynamics underscores the need for a holistic, nuanced approach to fostering genuine acceptance and integration of LGBTQ individuals within communities.

The absence of consistently significant treatment effects serves as a reflection of a preexisting landscape characterized by deeply entrenched anti-LGBTQ sentiments. This pattern aligns with the recurrent findings evident across multiple waves of Afrobarometer data, spanning numerous African countries, Ghana included. It is within this well-established context of prevailing attitudes that we must situate our analysis of the null outcomes.

The largely null results also effectively shed light on the formidable challenges that civil organizations and pro-LGBTQ groups encounter within the Ghanaian socio-cultural milieu. They reveal the uphill battle these entities face in their endeavors to transform and shift public perceptions. The documented patterns of staunch opposition to LGBTQ rights and the hesitance to embrace LGBTQ individuals within communities paint a complex backdrop against which these organizations operate. This nuanced interpretation underscores the necessity for a comprehensive understanding of the broader landscape and the complex dynamics at play. While the immediate treatment effects might not attain the desired level of statistical significance, they mirror the formidable hurdles that pro-LGBTQ advocacy groups confront. These challenges are deeply rooted in the bedrock of societal norms, historical contexts, and prevalent attitudes. The null results, in this context, offer a critical juncture for reflection and recalibration of strategies to effect meaningful change in the face of deeply ingrained resistance.

## Conclusion

In this study, we present findings that highlight the varying impacts of endorsement messages from local traditional, co-partisan political, and religious figures on the attitudes towards the LGBTQ community in Ghanaian society. Our results indicate that appeals from traditional leaders and co-partisan political leaders can moderately influence attitude shifts. We observe that messaging from religious leaders, in contrast, does not exhibit the same effectiveness. However, it is crucial to recognize that the extent of these effects is notably limited. Specifically, our analysis does not reveal any noteworthy impacts of these trusted authority endorsements on other dependent variables. These variables encompass a range of attitudes, including sympathizing with the challenges faced by the LGBTQ community, engaging in business interactions with LGBTQ individuals, supporting pro-LGBTQ candidates through voting, and accepting LGBTQ persons as residents within respondents’ communities.

While the endorsement cues succeed in sparking incremental change, the crux of the matter rests in recognizing the limitations inherent within this change in the short term. The intrinsic fabric of societal norms, preconceived notions, and deeply ingrained perspectives sets a formidable backdrop that ultimately constrains the extent to which authority elite cues can reshape attitudes. This complex interplay underscores that while the cues certainly wield influence by shifting opinions on LGBTQ rights up to seven percent, they grapple with the preexisting edifice of beliefs that have stood the test of time.

In a sense, this study stands as a testament to the delicate balance between the power of persuasion and the resilience of deeply rooted convictions. The endorsement messages effectively open a space for reconsideration and introspection, yet the journey toward meaningful and transformative change remains a complex endeavor. In this context, we contend that endorsement cues can cause shifts in attitudes. While this is useful, it also has some limits that must be taken into account in terms of policy.

## Supporting information

S1 Fig Support for LGTBQ across Africa. (TIF)

S2 Fig Experimental design. (TIF)

S3 Fig Support for LGBTQ rights (Logit). (TIF)

S4 Fig Support for pro-LGBTQ political candidates (Logit). (TIF)

S5 Fig Willingness to do business with members of the LGBTQ community (Logit). (TIF)

S6 Fig Probability of supporting LGBTQ rights. (TIF)

S7 Fig Probability of supporting pro-LGBTQ candidates. (TIF)

S8 Fig Probability of doing business with LGBTQ members and communities. (TIF)

S1 File The supporting information file provides further details about the experiment design, including demographics, tables with results and interactions, balance checks, and the survey instrument. (DOCX)

S2 File Dataset and replication code. https://figshare.com/articles/dataset/Dataset_for_Replication_Code_file_do-file_/25222178.
(ZIP)
