# Supplementary figures and images for: Can trusted authorities change minds on anti-LGBTQ norms: Evidence from an experiment in Ghana
Source: PLoS One. 2024 Aug 22;19(8):e0304698. doi: 10.1371/journal.pone.0304698 (PMC11341031; doi:10.1371/journal.pone.0304698)

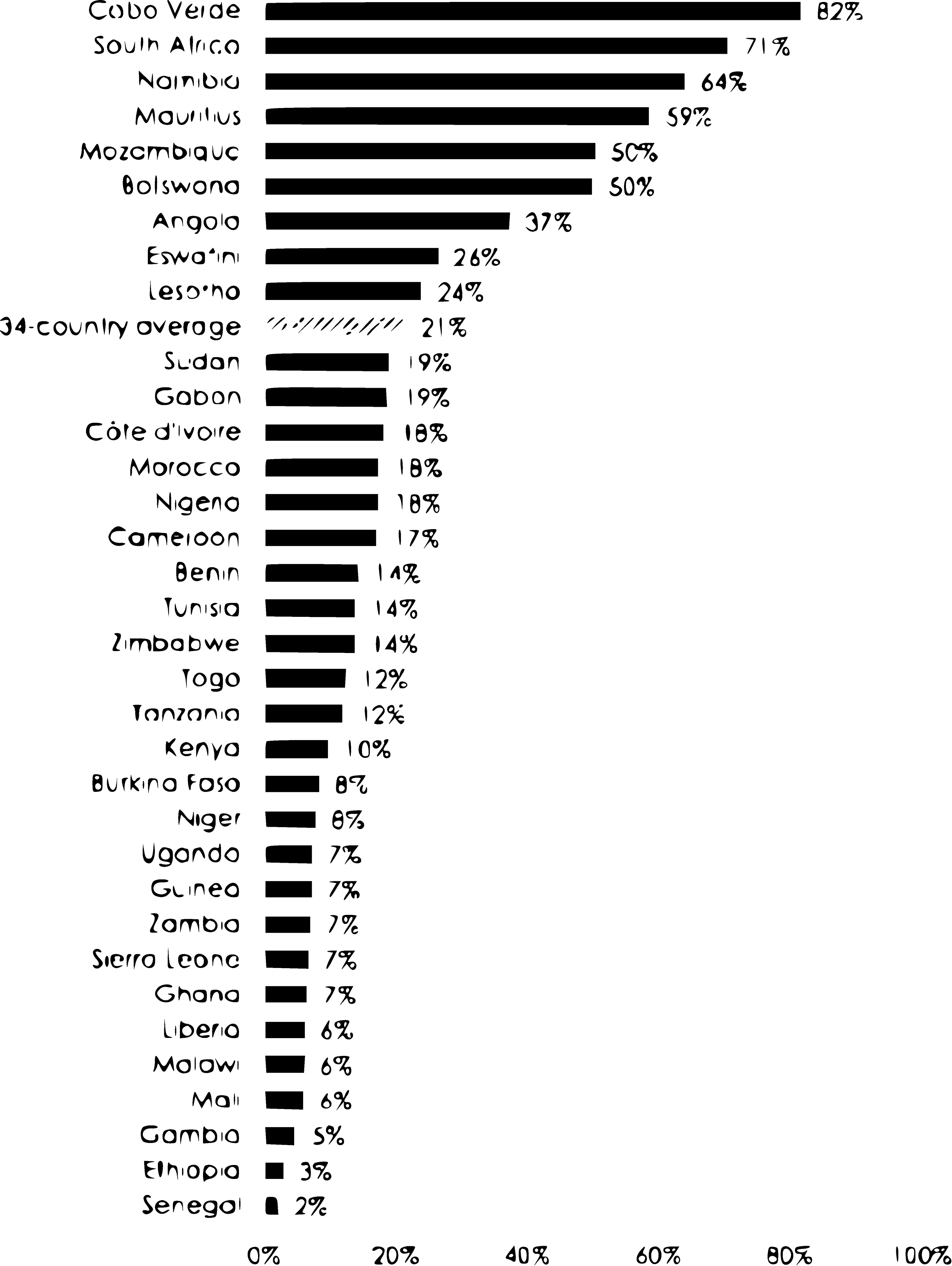

Supplement: S1 Fig — (TIF) [file pone.0304698.s001.tif]

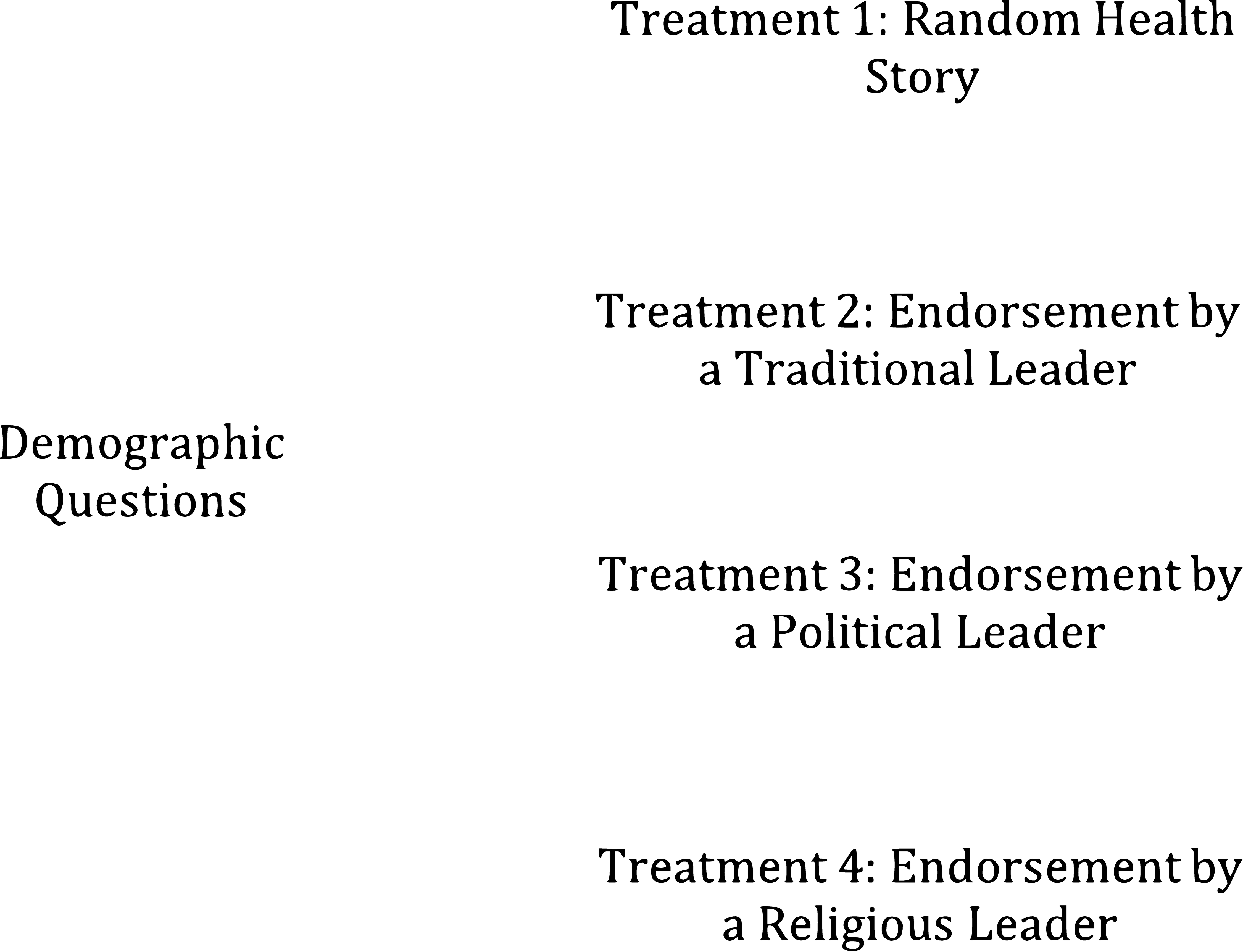

Supplement: S2 Fig — (TIF) [file pone.0304698.s002.tif]

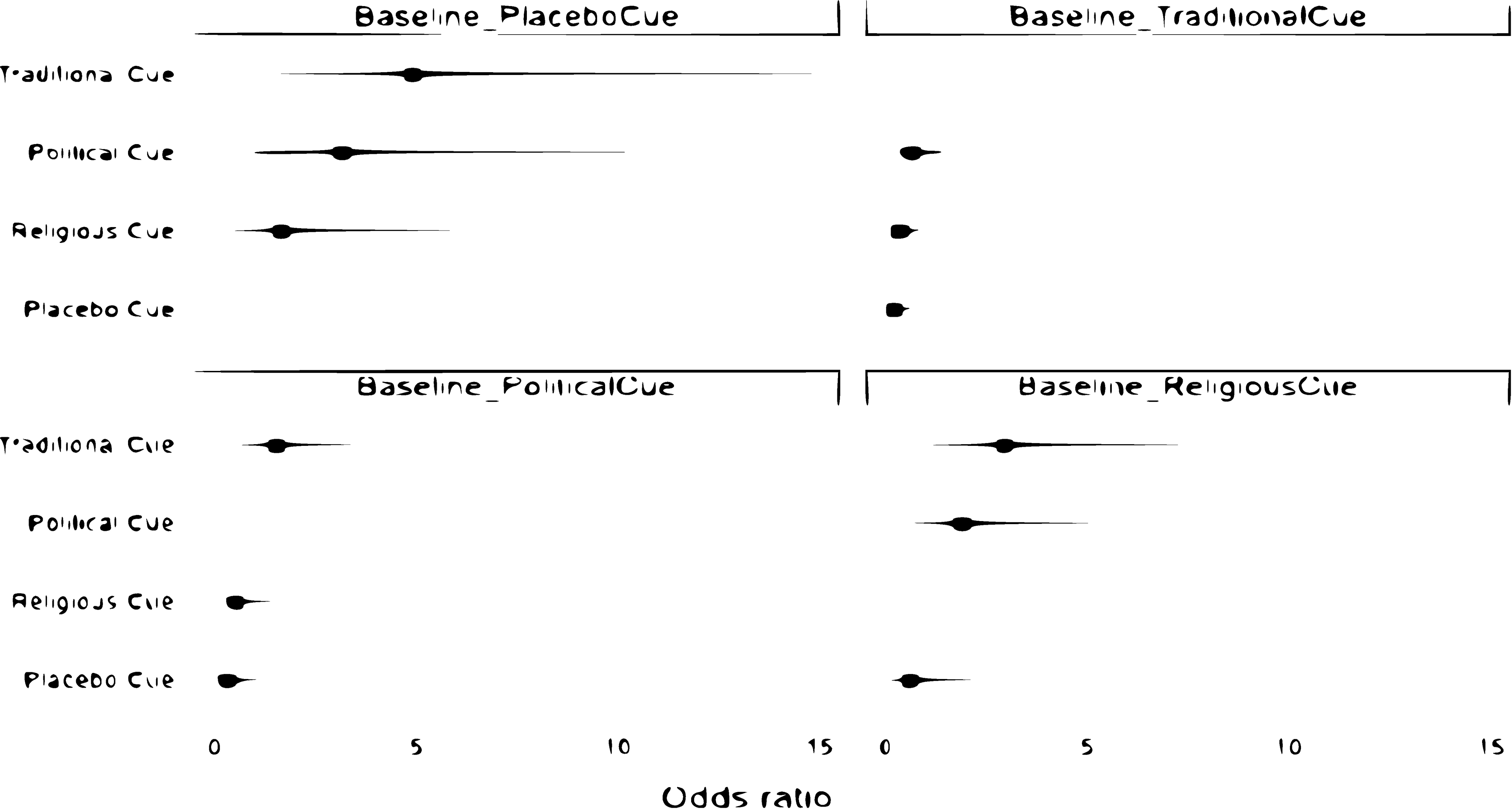

Supplement: S3 Fig — (TIF) [file pone.0304698.s003.tif]

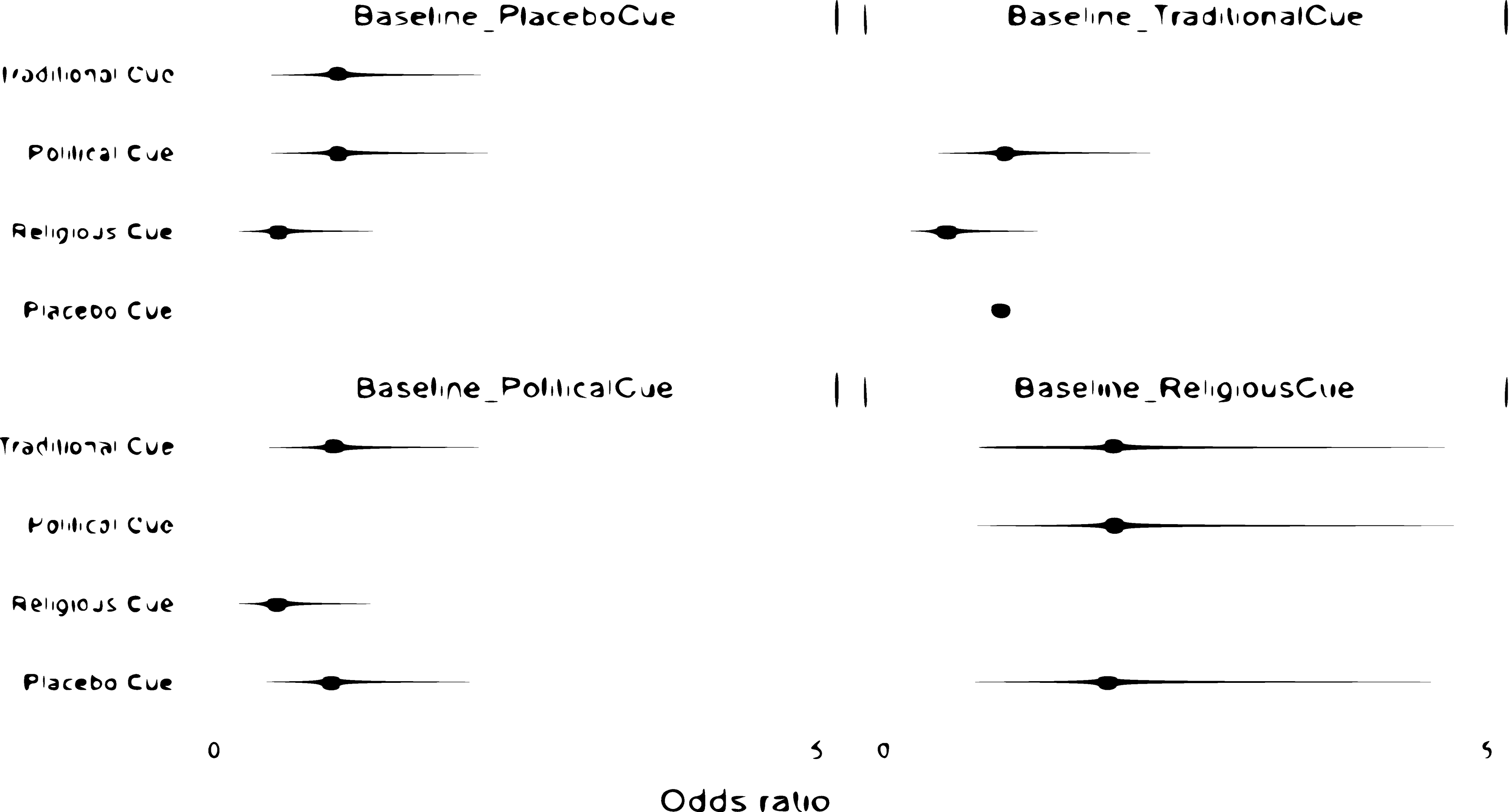

Supplement: S4 Fig — (TIF) [file pone.0304698.s004.tif]

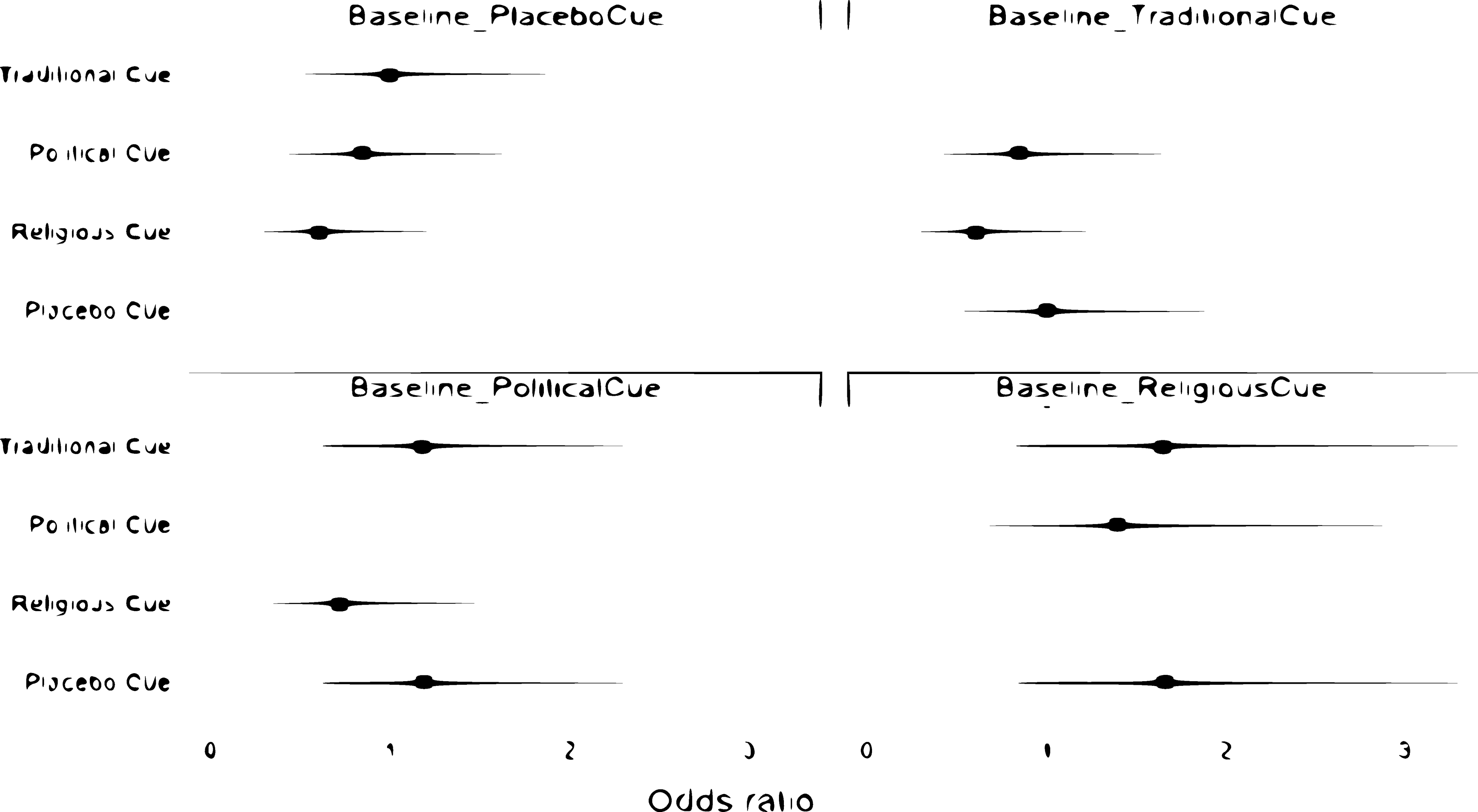

Supplement: S5 Fig — (TIF) [file pone.0304698.s005.tif]

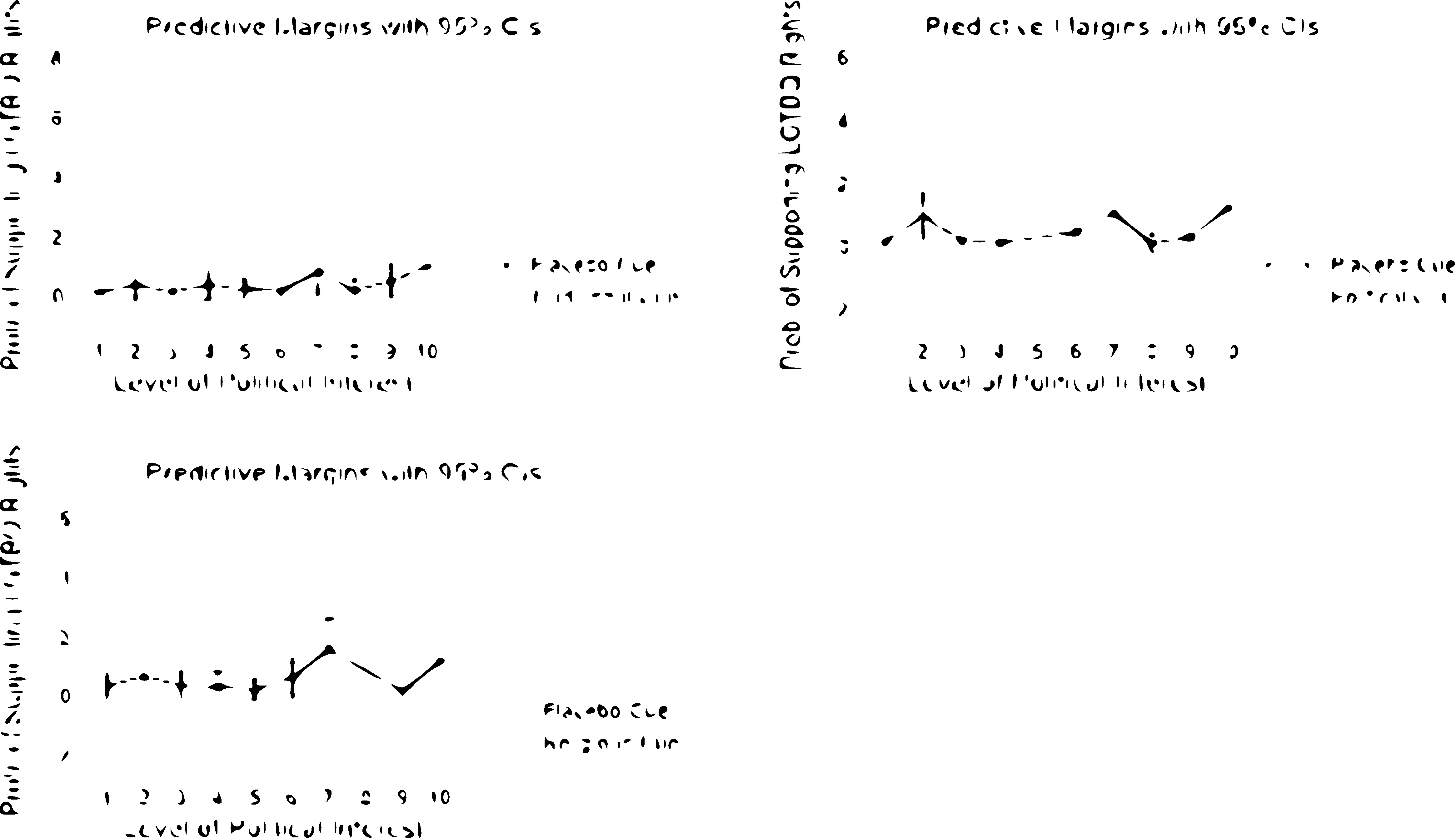

Supplement: S6 Fig — (TIF) [file pone.0304698.s006.tif]

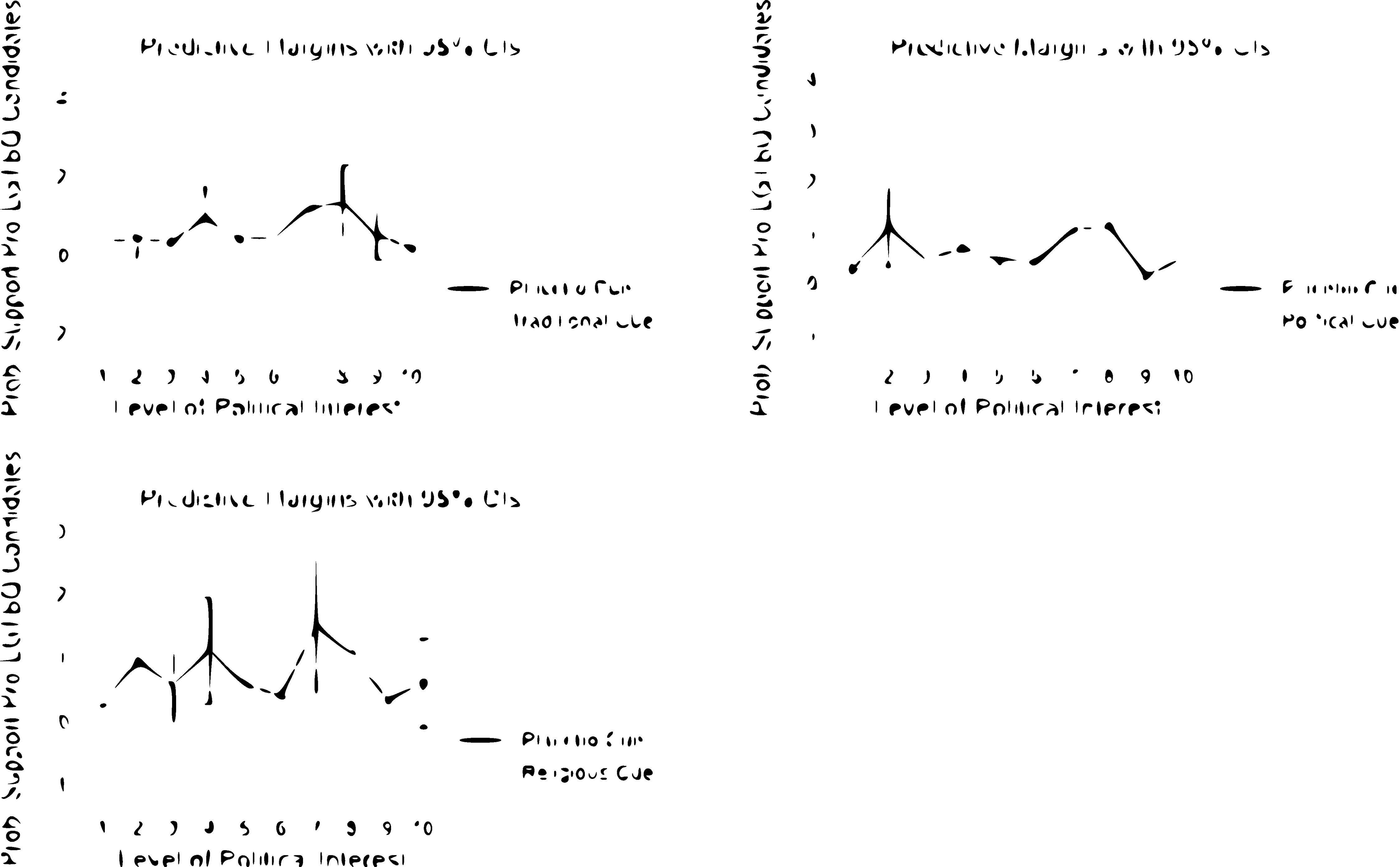

Supplement: S7 Fig — (TIF) [file pone.0304698.s007.tif]

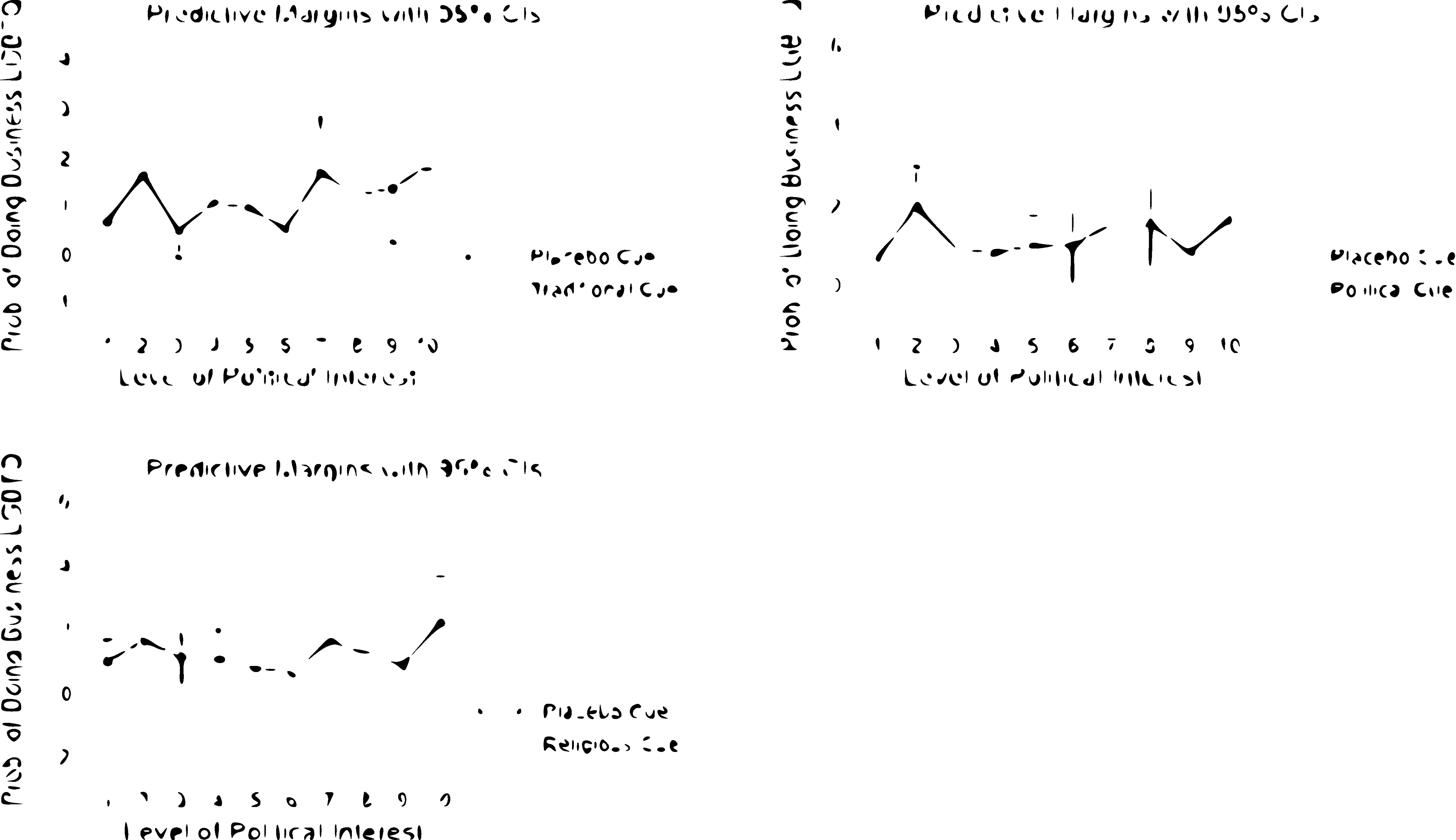

Supplement: S8 Fig — (TIF) [file pone.0304698.s008.tif]
