# Supplementary material for: Can trusted authorities change minds on anti-LGBTQ norms: Evidence from an experiment in Ghana
Source: PLoS One. 2024 Aug 22;19(8):e0304698. doi: 10.1371/journal.pone.0304698 (PMC11341031; doi:10.1371/journal.pone.0304698)
Supplement: S1 File — (DOCX) [file pone.0304698.s009.docx]

**S1 Supporting Information**

# Table of Contents

[Sample Design and Selection 1](#_Toc159182891)

[Main Results Tables 7](#_Toc159182892)

[Main Results Tables with Interactions 14](#_Toc159182893)

[Balance Checks 29](#_Toc159182894)

[Survey Instrument 31](#_Toc159182895)

[References 44](#_Toc159182896)

[Inclusivity in global research 45](#_Toc159182897)

# Sample Design and Selection

This study was designed as a cross-section and representative nationwide sample survey undertaken in Ghana. Ghana has 16 administrative regions and 261 districts. We combined probability and non-probability sampling techniques to ensure equal distribution of urban and rural districts where the respondents were selected. First, all 16 administrative regions were represented in the sample selection. A stratified multistage random sampling technique was then adopted to select the respondents from the regions. For the purposes of stratification, the region was stratified into urban and rural districts, from which two districts were selected. The district of the regional capitals was purposely selected to represent urban areas, but the district of the rural areas was randomly selected using random numbers.

The second stage involved the selection of dwelling structures. Random route walks with fixed sampling intervals (every 3^rd^ and 5^th^ house in rural and urban areas) were used at this stage in the selection of dwelling structures or houses. The primary sampling unit was the household in the towns and cities of the selected districts. When the enumerator entered a house, he or she counted the number of households within the house. Where there was more than one eligible respondent in a household, the enumerator used a lottery method to select one household. Every household in Ghana had an equal chance of inclusion in the sample. A household, for the purpose of this survey follows that of Ghana Statistical Service (2000), which defines it a person or group of persons who live together in the same house or compound, share the same housekeeping arrangements, and are catered for as one unit.

The quota for the two largest and most populated regions was doubled to account for proportional representation. The sample allocation to the rest of the regions was an equal proportion. The respondents were selected randomly amongst the males and females in the household until the quota of the selected area was achieved. Only one respondent from each household was selected. The survey sample size and distribution are captured in Table 1 below.

**Table 5: Sample Characteristics**

| Variables | Values | Percentages - Study Sample |
| --- | --- | --- |
| Age | 18-29  30-39  40-49  50-59  60+ | 3.40  32.3  19.8  10.5  3.30 |
| Ethnicity | Akan  Mole-Dagbon  Ewe  Ga-Adangbe  Konkomba  Guan  Gurunsi  Kusasi  Bissa  Other | 3.8  13.7  15.7  4.6  2.7  5.2  1.6  2.2  <1.0  18.6 |
| Political Affiliation | New Patriotic Party (NPP) | 25.6 |
|  | National Democratic Congress (NDC) | 29.6 |
|  | Convention People's Party (CPP) Peoples | 4.15 |
|  | National Convention (PNC) | 2.0 |
|  | Progressive People’s Party (PPP) | 1.8 |
|  | National Democratic Party (NDP) | <1.0 |
|  | Other | 3.2 |
|  | Not Affiliated with any party | 32.8 |
| Religion | Christianity  Islam  Traditional  None/Non-religious  Other  Prefer not to answer | 68.0  22.6  4.1  3.2  1.0  2.0 |
|  |  |  |
| Gender | Male  Female | 49  51 |
| Residence | Urban  Rural | 60.0  40.0 |

Note: number of respondents = 821

**Table 6: Sample Distribution across the Regions in Ghana**

| Code | Region | Population | Urban | Rural | Sample Percentage |
| --- | --- | --- | --- | --- | --- |
| 1 | Western Region | 31 | 30 | 1 | 3.78 |
| 2 | Western North | 44 | 21 | 23 | 5.36 |
| 3 | Central Region | 43 | 35 | 8 | 5.24 |
| 4 | Greater Accra Region | 108 | 107 | 1 | 13.15 |
| 5 | Volta Region | 46 | 20 | 26 | 5.60 |
| 6 | Oti Region | 44 | 22 | 22 | 5.36 |
| 7 | Eastern Region | 63 | 39 | 24 | 7.67 |
| 8 | Ashanti Region | 84 | 43 | 41 | 10.23 |
| 9 | Bono Region | 45 | 17 | 28 | 5.48 |
| 10 | Bono East Region | 40 | 19 | 21 | 4.87 |
| 11 | Ahafo Region | 43 | 20 | 23 | 5.24 |
| 12 | Northern Region | 43 | 26 | 17 | 5.24 |
| 13 | Savana Region | 47 | 25 | 22 | 5.72 |
| 14 | Northeast Region | 53 | 26 | 27 | 6.46 |
| 15 | Upper East Region | 40 | 15 | 25 | 4.87 |
| 16 | Upper West Region | 47 | 25 | 22 | 5.72 |
|  | Totals | 821 | 492 | 329 | 100 |

A One-day training session was organized for all the 32 enumerators. The training was done via zoom on 23^rd^ April 2023. The training took the form of lectures and discussions. The background and the objectives of the study were outlined. Officers were taken through the research instruments; the questionnaire and the import of each question was reiterated. This was followed with a demonstration of how the questionnaire should be administered. They also agreed on translation of certain words and phrases that would be used in the field. Essentially, the target population for the study was defined and the research assistants were taught how to select the household and the final respondents. Each research assistant followed the procedure of household and individual selection and administered two questionnaires.

The fieldwork report was discussed together, and this indeed deepened their understanding of the process. Field work started on the 24^th of^ April 2023 and ended on 15^th^ May 2023. Administration of the questionnaire was done in the language of the respondent’s choice. They were also tasked to administer six questionnaires on average for a day. In order to ensure reliability and accuracy of the results, the following quality assurance procedures were followed. We selected trained and experienced research assistants who have worked in other similar data collection projects. Daily reviews of completed questionnaires for legibility, accuracy, and consistency, monitored accuracy of individual interviews, and field spot checks of interviewers’ work.

**Survey Instrument and Variable Operationalizations**

The questionnaire evolved out of a series of meetings with principal research officers and the relevant literature on public opinion and LGBTQ issues. Research Officers carried out a pre-testing of a questionnaire for three days with students from the University of Ghana. Responses from the pre-test were used to refine the survey instruments. The instrument comprised seven sections. The questionnaire was designed to collect information on the background of the respondents with respect to sex, and place of residence.

The political interest variable is a simple additive index constructed from several other variables using a five point scale with the following categories: Never, One Time, Sometimes, Several Times, and On a Regular Basis. The categories Do not know and Refuse to answer were included as options but are not used in the construction of the index or its analysis. Furthermore, the pre-treatment questions capture respondents’ gender, region, urban-rural status, political affiliation, and ethnicity.

**Ethical Considerations**

Our main priority in the study was to ensure that our enumerators follow the required safety and ethical standards and because of that the few procedures were followed. First, we provided and read consent forms to the participants and explained the procedure of the experiment, including the nature of the questions. We worked closely with local researchers to navigate the local communities in the most safe and discrete manner. Second, this study employed deception as the messages we used were not in fact made by the respective authority figures. After the experiment, the enumerators debriefed the participants and explained to them that the messages were not real and that we deployed deception to make the treatments appear realistic. To the best of our knowledge the study did not generate threats to our participants, enumerators, and supervisors.

# Main Results Tables

Table 7: Support for Rights for the LGTBQ individuals and community No Controls

|  | (1) | (2) | (3) | (4) |
| --- | --- | --- | --- | --- |
|  | Placebo Cue | Traditional Cue | Political Cue | Religious Cue |
|  |  |  |  |  |
| Traditional_Cue | 4.919*** |  | 1.551 | 2.930** |
|  | (2.768) |  | (0.617) | (1.341) |
| Political_Cue | 3.172* | 0.645 |  | 1.890 |
|  | (1.883) | (0.257) |  | (0.935) |
| Religious_Cue | 1.679 | 0.341** | 0.529 |  |
|  | (1.066) | (0.156) | (0.262) |  |
| Placebo_Cue |  | 0.203*** | 0.315* | 0.596 |
|  |  | (0.114) | (0.187) | (0.378) |
| Constant | 0.021*** | 0.105*** | 0.067*** | 0.036*** |
|  | (0.011) | (0.026) | (0.021) | (0.014) |
|  |  |  |  |  |
| Observations | 759 | 759 | 759 | 759 |
| Pseudo R-squared | 0.0396 | 0.0396 | 0.0396 | 0.0396 |

Standard Errors in parentheses

*** p<0.01, ** p<0.05, * p<0.1

Table 8: Support for Pro-LGTBQ Candidates No Controls

|  | (1) | (2) | (3) | (4) |
| --- | --- | --- | --- | --- |
|  | Placebo Cue | Traditional Cue | Political Cue | Religious Cue |
|  |  |  |  |  |
| Traditional_Cue | 1.028 |  | 0.998 | 2.181 |
|  | (0.404) |  | (0.399) | (1.035) |
| Political_Cue | 1.030 | 1.002 |  | 2.186 |
|  | (0.412) | (0.401) |  | (1.051) |
| Religious_Cue | 0.472 | 0.459 | 0.458 |  |
|  | (0.224) | (0.218) | (0.220) |  |
| Placebo_Cue |  | 0.973 | 0.970 | 2.121 |
|  |  | (0.382) | (0.388) | (1.006) |
| Constant | 0.077*** | 0.079*** | 0.079*** | 0.036*** |
|  | (0.021) | (0.022) | (0.023) | (0.014) |
|  |  |  |  |  |
| Observations | 764 | 764 | 764 | 764 |
| Pseudo R-squared | 0.0112 | 0.0112 | 0.0112 | 0.0112 |

Standard Errors in parentheses

*** p<0.01, ** p<0.05, * p<0.1

Table 9: Doing business with members of the LGBTQ community No Controls

|  | (1) | (2) | (3) | (4) |
| --- | --- | --- | --- | --- |
|  | Placebo Cue | Traditional Cue | Political Cue | Religious Cue |
|  |  |  |  |  |
| Traditional_Cue | 0.994 |  | 1.182 | 1.639 |
|  | (0.317) |  | (0.400) | (0.578) |
| Political_Cue | 0.841 | 0.846 |  | 1.386 |
|  | (0.281) | (0.286) |  | (0.509) |
| Religious_Cue | 0.606 | 0.610 | 0.721 |  |
|  | (0.212) | (0.215) | (0.265) |  |
| Placebo_Cue |  | 1.006 | 1.189 | 1.649 |
|  |  | (0.321) | (0.398) | (0.577) |
| Constant | 0.145*** | 0.144*** | 0.122*** | 0.088*** |
|  | (0.032) | (0.033) | (0.030) | (0.024) |
|  |  |  |  |  |
| Observations | 709 | 709 | 709 | 709 |
| Pseudo R-squared | 0.00548 | 0.00548 | 0.00548 | 0.00548 |

Standard Errors in parentheses

*** p<0.01, ** p<0.05, * p<0.1

Table 10: Living in the community with LGBTQ member No Controls

|  | (1) | (2) | (3) | (4) |
| --- | --- | --- | --- | --- |
|  | Placebo Cue | Traditional Cue | Political Cue | Religious Cue |
|  |  |  |  |  |
| Traditional_Cue | 1.028 |  | 0.998 | 2.181 |
|  | (0.404) |  | (0.399) | (1.035) |
| Political_Cue | 1.030 | 1.002 |  | 2.186 |
|  | (0.412) | (0.401) |  | (1.051) |
| Religious_Cue | 0.472 | 0.459 | 0.458 |  |
|  | (0.224) | (0.218) | (0.220) |  |
| Placebo_Cue |  | 0.973 | 0.970 | 2.121 |
|  |  | (0.382) | (0.388) | (1.006) |
| Constant | 0.077*** | 0.079*** | 0.079*** | 0.036*** |
|  | (0.021) | (0.022) | (0.023) | (0.014) |
|  |  |  |  |  |
| Observations | 764 | 764 | 764 | 764 |
| Pseudo R-squared | 0.0112 | 0.0112 | 0.0112 | 0.0112 |

Standard Errors in parentheses

*** p<0.01, ** p<0.05, * p<0.1

The outcomes presented in Table 10 follow a similar trend of limited treatment effects, with a couple of notable exceptions. Roughly 28 percent of respondents exposed to the placebo health story express willingness to reside in communities with LGBTQ members, while this number drops to 19.4 percent for those encountering an endorsement cue from local traditional leaders. Similarly, those treated with an endorsement cue from a co-partisan political leader show a willingness of 20 percent, lower than the 28 percent in the placebo group. It's important to note that these results achieve statistical significance at a 90 percent confidence level. However, comparisons between cues from traditional, political, or religious leaders do not yield significant mean differences. Overall, support for living in communities with LGBTQ members ranges from 19.4 to 28 percent. This lack of consistent treatment effects is a crucial observation and is explored further in the discussion section.

Table 11: Self-Reported Understanding of the problems that the LGBTQ community is facing in the country No Controls.

|  | (1) | (2) | (3) | (4) |
| --- | --- | --- | --- | --- |
|  | Placebo Cue | Traditional Cue | Political Cue | Religious Cue |
|  |  |  |  |  |
| Traditional_Cue | 1.000 |  | 0.829 | 1.155 |
|  | (0.310) |  | (0.256) | (0.365) |
| Political_Cue | 1.206 | 1.206 |  | 1.393 |
|  | (0.373) | (0.373) |  | (0.438) |
| Religious_Cue | 0.866 | 0.866 | 0.718 |  |
|  | (0.274) | (0.274) | (0.226) |  |
| Placebo_Cue |  | 1.000 | 0.829 | 1.155 |
|  |  | (0.310) | (0.256) | (0.365) |
| Constant | 0.156*** | 0.156*** | 0.188*** | 0.135*** |
|  | (0.034) | (0.034) | (0.041) | (0.031) |
|  |  |  |  |  |
| Observations | 699 | 699 | 699 | 699 |
| Pseudo R-squared | 0.00201 | 0.00201 | 0.00201 | 0.00201 |

Standard Errors in parentheses

*** p<0.01, ** p<0.05, * p<0.1

Examining Table 10 outlining respondents' self-reported understanding of the difficulties of the LGBTQ community, it becomes apparent that there is no substantial or consistent impact resulting from different types of endorsement cues. For instance, among respondents exposed to the placebo health story, only 13 percent express an understanding of LGBTQ community challenges, while those informed about the endorsement message from a local traditional leader show a slight increase at 13.4 percent. Conversely, exposure to an endorsement message from a political leader raised understanding to 16 percent, compared to 12 percent for the religious leader’s treatment. However, the 4 percent difference lacks statistical significance due to considerable variance among the groups. Remarkably, approximately 80 percent of respondents lack an understanding of challenges of LGBTQ community, which serves as an indicative proxy for their limited willingness to engage with these concerns. As such, hypothesis two (H2) and its variations are not substantiated.

Table 12: Support for Rights for the LGTBQ individuals and community with Controls

|  | Baseline: | Baseline: | Baseline: | Baseline: |
| --- | --- | --- | --- | --- |
|  | Placebo Cue | Traditional Cue | Political Cue | Religious Cue |
|  |  |  |  |  |
| Traditional_Cue | 4.654*** |  | 1.493 | 2.826** |
|  | (2.651) |  | (0.606) | (1.314) |
| Political_Cue | 3.118* | 0.670 |  | 1.893 |
|  | (1.862) | (0.272) |  | (0.943) |
| Religious_Cue | 1.647 | 0.354** | 0.528 |  |
|  | (1.049) | (0.165) | (0.263) |  |
| Age | 1.007 | 1.007 | 1.007 | 1.007 |
|  | (0.160) | (0.160) | (0.160) | (0.160) |
| Political_Interest | 1.110 | 1.110 | 1.110 | 1.110 |
|  | (0.072) | (0.072) | (0.072) | (0.072) |
| Gender | 1.330 | 1.330 | 1.330 | 1.330 |
|  | (0.455) | (0.455) | (0.455) | (0.455) |
| Ethnicity | 0.988 | 0.988 | 0.988 | 0.988 |
|  | (0.030) | (0.030) | (0.030) | (0.030) |
| Political_party | 0.975 | 0.975 | 0.975 | 0.975 |
|  | (0.021) | (0.021) | (0.021) | (0.021) |
| Placebo Cue |  | 0.215*** | 0.321* | 0.607 |
|  |  | (0.122) | (0.192) | (0.387) |
| Constant | 0.012*** | 0.055*** | 0.037*** | 0.020*** |
|  | (0.011) | (0.046) | (0.032) | (0.017) |
|  |  |  |  |  |
| Observations | 733 | 733 | 733 | 733 |
| Pseudo R-squared | 0.0552 | 0.0552 | 0.0552 | 0.0552 |

Standard Errors in parentheses

*** p<0.01, ** p<0.05, * p<0.1

Table 13: Support for pro-LGTBQ candidates with Controls

|  | Baseline: | Baseline: | Baseline: | Baseline: |
| --- | --- | --- | --- | --- |
|  | Placebo Cue | Traditional Cue | Political Cue | Religious Cue |
|  |  |  |  |  |
| Traditional_Cue | 1.088 |  | 0.992 | 2.263* |
|  | (0.431) |  | (0.402) | (1.085) |
| Political_Cue | 1.060 | 1.008 |  | 2.281* |
|  | (0.427) | (0.408) |  | (1.107) |
| Religious_Cue | 0.480 | 0.442* | 0.438* |  |
|  | (0.228) | (0.212) | (0.213) |  |
| Age | 1.014 | 1.007 | 1.007 | 1.007 |
|  | (0.060) | (0.060) | (0.060) | (0.060) |
| Political_Interest | 1.015 | 1.043 | 1.043 | 1.043 |
|  | (0.151) | (0.156) | (0.156) | (0.156) |
| Gender | 1.016 | 0.992 | 0.992 | 0.992 |
|  | (0.310) | (0.304) | (0.304) | (0.304) |
| Ethnicity | 1.016 | 1.022 | 1.022 | 1.022 |
|  | (0.027) | (0.027) | (0.027) | (0.027) |
| Political_party | 0.963* | 0.959** | 0.959** | 0.959** |
|  | (0.019) | (0.019) | (0.019) | (0.019) |
| Placebo Cue |  | 0.927 | 0.920 | 2.099 |
|  |  | (0.369) | (0.373) | (1.002) |
| Constant | 0.095*** | 0.013*** | 0.013*** | 0.006*** |
|  | (0.071) | (0.016) | (0.016) | (0.007) |
|  |  |  |  |  |
| Observations | 737 | 737 | 737 | 737 |
| Pseudo R-squared | 0.0225 | 0.0381 | 0.0381 | 0.0381 |

Standard Errors in parentheses

*** p<0.01, ** p<0.05, * p<0.1

Table 14: Doing business with members of the LGBTQ community with controls.

|  | Baseline: | Baseline: | Baseline: | Baseline: |
| --- | --- | --- | --- | --- |
|  | Placebo Cue | Traditional Cue | Political Cue | Religious Cue |
|  |  |  |  |  |
| Traditional_Cue | 1.025 |  | 1.160 | 1.597 |
|  | (0.334) |  | (0.396) | (0.569) |
| Political_Cue | 0.884 | 0.862 |  | 1.377 |
|  | (0.301) | (0.294) |  | (0.509) |
| Religious_Cue | 0.642 | 0.626 | 0.726 |  |
|  | (0.228) | (0.223) | (0.269) |  |
| Age | 1.044 | 1.044 | 1.044 | 1.044 |
|  | (0.051) | (0.051) | (0.051) | (0.051) |
| Political_Interest | 0.754** | 0.754** | 0.754** | 0.754** |
|  | (0.100) | (0.100) | (0.100) | (0.100) |
| Gender | 1.018 | 1.018 | 1.018 | 1.018 |
|  | (0.254) | (0.254) | (0.254) | (0.254) |
| Ethnicity | 0.978 | 0.978 | 0.978 | 0.978 |
|  | (0.022) | (0.022) | (0.022) | (0.022) |
| Political_party | 1.019 | 1.019 | 1.019 | 1.019 |
|  | (0.018) | (0.018) | (0.018) | (0.018) |
| Placebo Cue |  | 0.975 | 1.131 | 1.557 |
|  |  | (0.318) | (0.385) | (0.553) |
| Constant | 0.187*** | 0.192*** | 0.165*** | 0.120*** |
|  | (0.115) | (0.118) | (0.105) | (0.076) |
|  |  |  |  |  |
| Observations | 683 | 683 | 683 | 683 |
| Pseudo R-squared | 0.0197 | 0.0197 | 0.0197 | 0.0197 |

Standard Errors in parentheses

*** p<0.01, ** p<0.05, * p<0.1

Table 15: Living in the community with LGBTQ member with Controls.

|  | Baseline: | Baseline: | Baseline: | Baseline: |
| --- | --- | --- | --- | --- |
|  | Placebo Cue | Traditional Cue | Political Cue | Religious Cue |
|  |  |  |  |  |
| Traditional_Cue | 1.088 |  | 1.027 | 2.268* |
|  | (0.431) |  | (0.413) | (1.083) |
| Political_Cue | 1.060 | 0.974 |  | 2.209 |
|  | (0.427) | (0.392) |  | (1.068) |
| Religious_Cue | 0.480 | 0.441* | 0.453 |  |
|  | (0.228) | (0.211) | (0.219) |  |
| Age | 1.014 | 1.014 | 1.014 | 1.014 |
|  | (0.060) | (0.060) | (0.060) | (0.060) |
| Political_Interest | 1.015 | 1.015 | 1.015 | 1.015 |
|  | (0.151) | (0.151) | (0.151) | (0.151) |
| Gender | 1.016 | 1.016 | 1.016 | 1.016 |
|  | (0.310) | (0.310) | (0.310) | (0.310) |
| Ethnicity | 1.016 | 1.016 | 1.016 | 1.016 |
|  | (0.027) | (0.027) | (0.027) | (0.027) |
| Political_party | 0.963* | 0.963* | 0.963* | 0.963* |
|  | (0.019) | (0.019) | (0.019) | (0.019) |
| Placebo Cue |  | 0.919 | 0.944 | 2.084 |
|  |  | (0.364) | (0.380) | (0.992) |
| Constant | 0.095*** | 0.103*** | 0.100*** | 0.045*** |
|  | (0.071) | (0.078) | (0.077) | (0.036) |
|  |  |  |  |  |
| Observations | 737 | 737 | 737 | 737 |
| Pseudo R-squared | 0.0225 | 0.0225 | 0.0225 | 0.0225 |

Standard Errors in parentheses

*** p<0.01, ** p<0.05, * p<0.1

Table 16: Self-Reported Understanding of the problems that the LGBTQ community is facing in the country with Controls.

|  | Baseline: | Baseline: | Baseline: | Baseline: |
| --- | --- | --- | --- | --- |
|  | Placebo Cue | Traditional Cue | Political Cue | Religious Cue |
|  |  |  |  |  |
| Traditional_Cue | 0.967 |  | 0.835 | 1.136 |
|  | (0.303) |  | (0.260) | (0.362) |
| Political_Cue | 1.158 | 1.198 |  | 1.360 |
|  | (0.361) | (0.373) |  | (0.431) |
| Religious_Cue | 0.851 | 0.881 | 0.735 |  |
|  | (0.271) | (0.281) | (0.233) |  |
| Age | 1.086* | 1.086* | 1.086* | 1.086* |
|  | (0.047) | (0.047) | (0.047) | (0.047) |
| Political_Interest | 0.942 | 0.942 | 0.942 | 0.942 |
|  | (0.105) | (0.105) | (0.105) | (0.105) |
| Gender | 1.104 | 1.104 | 1.104 | 1.104 |
|  | (0.250) | (0.250) | (0.250) | (0.250) |
| Ethnicity | 0.991 | 0.991 | 0.991 | 0.991 |
|  | (0.020) | (0.020) | (0.020) | (0.020) |
| Political_party | 1.014 | 1.014 | 1.014 | 1.014 |
|  | (0.016) | (0.016) | (0.016) | (0.016) |
| Placebo Cue |  | 1.035 | 0.864 | 1.175 |
|  |  | (0.324) | (0.269) | (0.373) |
| Constant | 0.094*** | 0.090*** | 0.108*** | 0.080*** |
|  | (0.053) | (0.052) | (0.063) | (0.046) |
|  |  |  |  |  |
| Observations | 676 | 676 | 676 | 676 |
| Pseudo R-squared | 0.00952 | 0.00952 | 0.00952 | 0.00952 |

Standard Errors in parentheses

*** p<0.01, ** p<0.05, * p<0.1

# Main Results Tables with Interactions

Table 17: Support for Rights for the LGTBQ individuals and community

|  | Baseline: |
| --- | --- |
|  | Placebo Cue |
|  |  |
| Traditional Cue | 11.011** |
|  | (10.457) |
| Political Interest | 1.205** |
|  | (0.107) |
| Traditional Cue*Political Interest | 0.818 |
|  | (0.107) |
| Political Cue | 2.632 |
|  | (1.603) |
| Religious Cue | 1.374 |
|  | (0.896) |
| Age | 1.178 |
|  | (0.194) |
| Gender | 1.265 |
|  | (0.458) |
| Ethnicity | 1.004 |
|  | (0.032) |
| Political Party | 0.968 |
|  | (0.023) |
| Constant | 0.222 |
|  | (0.271) |
|  |  |
| Observations | 729 |
| Pseudo R-squared | 0.144 |

Standard Errors in parentheses

*** p<0.01, ** p<0.05, * p<0.1

Table 18: Support for Rights for the LGTBQ individuals and community

|  | Baseline: |
| --- | --- |
|  | Traditional Cue |
|  |  |
| Placebo Cue | 0.071* |
|  | (0.108) |
| Political Interest | 1.090 |
|  | (0.074) |
| Placebo Cue*Political Interest | 1.192 |
|  | (0.247) |
| Political Cue | 0.674 |
|  | (0.273) |
| Religious Cue | 0.357** |
|  | (0.166) |
| Age | 1.013 |
|  | (0.161) |
| Gender | 1.315 |
|  | (0.450) |
| Ethnicity | 0.987 |
|  | (0.030) |
| Political Party | 0.976 |
|  | (0.021) |
| Constant | 0.062*** |
|  | (0.052) |
|  |  |
| Observations | 733 |
| Pseudo R-squared | 0.0578 |

Standard Errors in parentheses

*** p<0.01, ** p<0.05, * p<0.1

Table 19: Support for Rights for the LGTBQ individuals and community

|  | Baseline: |
| --- | --- |
|  | Political Cue |
|  |  |
| Placebo Cue | 0.320* |
|  | (0.191) |
| Traditional Cue | 1.480 |
|  | (0.600) |
| Religious Cue | 0.166 |
|  | (0.207) |
| Political Interest | 1.076 |
|  | (0.076) |
| Religious Cue*Political Interest | 1.196 |
|  | (0.198) |
| Age | 1.009 |
|  | (0.161) |
| Gender | 1.344 |
|  | (0.461) |
| Ethnicity | 0.988 |
|  | (0.029) |
| Political Party | 0.975 |
|  | (0.021) |
| Constant | 0.044*** |
|  | (0.038) |
|  |  |
| Observations | 733 |
| Pseudo R-squared | 0.0594 |

Standard Errors in parentheses

*** p<0.01, ** p<0.05, * p<0.1

Table 20: Support for Rights for the LGTBQ individuals and community

|  | Baseline: |
| --- | --- |
|  | Religious Cue |
|  |  |
| Placebo Cue | 0.607 |
|  | (0.387) |
| Traditional Cue | 2.821** |
|  | (1.313) |
| Political Cue | 1.732 |
|  | (1.630) |
| Political Interest | 1.106 |
|  | (0.081) |
| Political Cue*Political Interest | 1.015 |
|  | (0.135) |
| Age | 1.006 |
|  | (0.160) |
| Gender | 1.330 |
|  | (0.455) |
| Ethnicity | 0.988 |
|  | (0.030) |
| Political Party | 0.975 |
|  | (0.021) |
| Constant | 0.020*** |
|  | (0.018) |
|  |  |
| Observations | 733 |
| Pseudo R-squared | 0.0553 |

Standard Errors in parentheses

*** p<0.01, ** p<0.05, * p<0.1

Table 21: Support for pro-LGTBQ candidates

|  | Baseline: |
| --- | --- |
|  | Placebo Cue |
|  |  |
| Traditional Cue | 2.187 |
|  | (1.528) |
| Political Interest | 1.057 |
|  | (0.073) |
| Traditional Cue*Political Interest | 0.870 |
|  | (0.103) |
| Political Cue | 1.059 |
|  | (0.428) |
| Religious Cue | 0.476 |
|  | (0.227) |
| Age | 1.012 |
|  | (0.151) |
| Gender | 1.017 |
|  | (0.311) |
| Ethnicity | 1.016 |
|  | (0.027) |
| Political Party | 0.964* |
|  | (0.019) |
| Constant | 0.075*** |
|  | (0.058) |
|  |  |
| Observations | 737 |
| Pseudo R-squared | 0.0265 |

Standard Errors in parentheses

*** p<0.01, ** p<0.05, * p<0.1

Table 22: Support for pro-LGTBQ candidates

|  | Baseline: |
| --- | --- |
|  | Traditional Cue |
|  |  |
| Placebo Cue | 0.733 |
|  | (0.555) |
| Political Interest | 1.001 |
|  | (0.068) |
| Placebo Cue*Political Interest | 1.043 |
|  | (0.124) |
| Political Cue | 0.977 |
|  | (0.393) |
| Religious Cue | 0.443* |
|  | (0.212) |
| Age | 1.017 |
|  | (0.152) |
| Gender | 1.013 |
|  | (0.310) |
| Ethnicity | 1.015 |
|  | (0.027) |
| Political Party | 0.964* |
|  | (0.019) |
| Constant | 0.110*** |
|  | (0.085) |
|  |  |
| Observations | 737 |
| Pseudo R-squared | 0.0229 |

Standard Errors in parentheses

*** p<0.01, ** p<0.05, * p<0.1

Table 23: Support for pro-LGTBQ candidates

|  | Baseline: |
| --- | --- |
|  | Political Cue |
|  |  |
| Placebo Cue | 0.944 |
|  | (0.380) |
| Traditional Cue | 1.027 |
|  | (0.413) |
| Religious Cue | 0.448 |
|  | (0.420) |
| Political Interest | 1.013 |
|  | (0.064) |
| Religious Cue*Political Interest | 1.002 |
|  | (0.145) |
| Age | 1.015 |
|  | (0.151) |
| Gender | 1.016 |
|  | (0.310) |
| Ethnicity | 1.016 |
|  | (0.027) |
| Political Party | 0.963* |
|  | (0.019) |
| Constant | 0.101*** |
|  | (0.078) |
|  |  |
| Observations | 737 |
| Pseudo R-squared | 0.0225 |

Standard Errors in parentheses

*** p<0.01, ** p<0.05, * p<0.1

Table 24: Support for pro-LGTBQ candidates

|  | Baseline: |
| --- | --- |
|  | Religious Cue |
|  |  |
| Placebo Cue | 2.076 |
|  | (0.988) |
| Traditional Cue | 2.246* |
|  | (1.073) |
| Political Cue | 1.231 |
|  | (1.046) |
| Political Interest | 0.987 |
|  | (0.066) |
| Political Cue*Political Interest | 1.110 |
|  | (0.135) |
| Age | 1.007 |
|  | (0.151) |
| Gender | 1.021 |
|  | (0.312) |
| Ethnicity | 1.018 |
|  | (0.028) |
| Political Party | 0.964* |
|  | (0.019) |
| Constant | 0.052*** |
|  | (0.041) |
|  |  |
| Observations | 737 |
| Pseudo R-squared | 0.0246 |

Standard Errors in parentheses

*** p<0.01, ** p<0.05, * p<0.1

Table 25: Doing business with members of the LGBTQ community.

|  | Baseline: |
| --- | --- |
|  | Placebo Cue |
|  |  |
| Traditional Cue | 1.985 |
|  | (1.093) |
| Political Interest | 1.085 |
|  | (0.060) |
| Traditional Cue*Political Interest | 0.869 |
|  | (0.085) |
| Political Cue | 0.878 |
|  | (0.299) |
| Religious Cue | 0.636 |
|  | (0.226) |
| Age | 0.757** |
|  | (0.100) |
| Gender | 1.010 |
|  | (0.252) |
| Ethnicity | 0.978 |
|  | (0.022) |
| Political Party | 1.021 |
|  | (0.018) |
| Constant | 0.152*** |
|  | (0.097) |
|  |  |
| Observations | 683 |
| Pseudo R-squared | 0.0241 |

Standard Errors in parentheses

*** p<0.01, ** p<0.05, * p<0.1

Table 26: Doing business with members of the LGBTQ community.

|  | Baseline: |
| --- | --- |
|  | Traditional Cue |
|  |  |
| Placebo Cue | 0.623 |
|  | (0.368) |
| Political Interest | 1.017 |
|  | (0.057) |
| Placebo Cue*Political Interest | 1.093 |
|  | (0.105) |
| Political Cue | 0.870 |
|  | (0.297) |
| Religious Cue | 0.633 |
|  | (0.226) |
| Age | 0.757** |
|  | (0.100) |
| Gender | 1.005 |
|  | (0.251) |
| Ethnicity | 0.976 |
|  | (0.022) |
| Political Party | 1.020 |
|  | (0.018) |
| Constant | 0.219** |
|  | (0.137) |
|  |  |
| Observations | 683 |
| Pseudo R-squared | 0.0215 |

Standard Errors in parentheses

*** p<0.01, ** p<0.05, * p<0.1

Table 27: Doing business with members of the LGBTQ community.

|  | Baseline: |
| --- | --- |
|  | Political Cue |
|  |  |
| Placebo Cue | 1.129 |
|  | (0.385) |
| Traditional Cue | 1.156 |
|  | (0.395) |
| Religious Cue | 0.550 |
|  | (0.373) |
| Political Interest | 1.032 |
|  | (0.055) |
| Religious Cue*Political Interest | 1.054 |
|  | (0.111) |
| Age | 0.755** |
|  | (0.100) |
| Gender | 1.021 |
|  | (0.255) |
| Ethnicity | 0.978 |
|  | (0.022) |
| Political Party | 1.019 |
|  | (0.018) |
| Constant | 0.174*** |
|  | (0.112) |
|  |  |
| Observations | 683 |
| Pseudo R-squared | 0.0202 |

Standard Errors in parentheses

*** p<0.01, ** p<0.05, * p<0.1

Table 28: Doing business with members of the LGBTQ community.

|  | Baseline: |
| --- | --- |
|  | Religious Cue |
|  |  |
| Placebo Cue | 1.557 |
|  | (0.553) |
| Traditional Cue | 1.596 |
|  | (0.569) |
| Political Cue | 1.320 |
|  | (0.852) |
| Political Interest | 1.042 |
|  | (0.056) |
| Political Cue*Political Interest | 1.008 |
|  | (0.103) |
| Age | 0.754** |
|  | (0.100) |
| Gender | 1.018 |
|  | (0.254) |
| Ethnicity | 0.978 |
|  | (0.022) |
| Political Party | 1.019 |
|  | (0.018) |
| Constant | 0.121*** |
|  | (0.078) |
|  |  |
| Observations | 683 |
| Pseudo R-squared | 0.0197 |

Standard Errors in parentheses

*** p<0.01, ** p<0.05, * p<0.1

Table 29: Living in a community with LGBTQ members.

|  | Baseline: |
| --- | --- |
|  | Placebo Cue |
|  |  |
| Traditional Cue | 2.072 |
|  | (1.487) |
| Political Interest | 1.118* |
|  | (0.074) |
| Traditional Cue*Political Interest | 0.886 |
|  | (0.102) |
| Political Cue | 1.182 |
|  | (0.479) |
| Religious Cue | 0.733 |
|  | (0.320) |
| Age | 0.941 |
|  | (0.136) |
| Gender | 1.422 |
|  | (0.427) |
| Ethnicity | 0.986 |
|  | (0.026) |
| Political Party | 0.983 |
|  | (0.019) |
| Constant | 0.040*** |
|  | (0.030) |
|  |  |
| Observations | 694 |
| Pseudo R-squared | 0.0185 |

Standard Errors in parentheses

*** p<0.01, ** p<0.05, * p<0.1

Table 30: Living in a community with LGBTQ members.

|  | Baseline: |
| --- | --- |
|  | Traditional Cue |
|  |  |
| Placebo Cue | 0.327 |
|  | (0.280) |
| Political Interest | 1.034 |
|  | (0.067) |
| Placebo Cue*Political Interest | 1.197 |
|  | (0.148) |
| Political Cue | 1.100 |
|  | (0.436) |
| Religious Cue | 0.691 |
|  | (0.298) |
| Age | 0.951 |
|  | (0.137) |
| Gender | 1.410 |
|  | (0.424) |
| Ethnicity | 0.983 |
|  | (0.026) |
| Political Party | 0.983 |
|  | (0.019) |
| Constant | 0.067*** |
|  | (0.051) |
|  |  |
| Observations | 694 |
| Pseudo R-squared | 0.0213 |

Standard Errors in parentheses

*** p<0.01, ** p<0.05, * p<0.1

Table 31: Living in a community with LGBTQ members.

|  | Baseline: |
| --- | --- |
|  | Political Cue |
|  |  |
| Placebo Cue | 0.841 |
|  | (0.340) |
| Traditional Cue | 0.914 |
|  | (0.363) |
| Religious Cue | 0.534 |
|  | (0.457) |
| Political Interest | 1.075 |
|  | (0.068) |
| Religious Cue*Political Interest | 1.027 |
|  | (0.129) |
| Age | 0.941 |
|  | (0.136) |
| Gender | 1.430 |
|  | (0.429) |
| Ethnicity | 0.986 |
|  | (0.026) |
| Political Party | 0.983 |
|  | (0.019) |
| Constant | 0.059*** |
|  | (0.045) |
|  |  |
| Observations | 694 |
| Pseudo R-squared | 0.0155 |

Standard Errors in parentheses

*** p<0.01, ** p<0.05, * p<0.1

Table 32: Living in a community with LGBTQ members.

|  | Baseline: |
| --- | --- |
|  | Religious Cue |
|  |  |
| Placebo Cue | 1.356 |
|  | (0.592) |
| Traditional Cue | 1.471 |
|  | (0.635) |
| Political Cue | 2.297 |
|  | (1.788) |
| Political Interest | 1.098 |
|  | (0.071) |
| Political Cue*Political Interest | 1.000 |
|  | (0.000) |
| Age | 0.938 |
|  | (0.111) |
| Gender | 0.944 |
|  | (0.136) |
| Ethnicity | 1.430 |
|  | (0.429) |
| Political Party | 0.985 |
|  | (0.026) |
| Placebo Cue | 0.982 |
|  | (0.019) |
| Constant | 0.033*** |
|  | (0.025) |
|  |  |
| Observations | 694 |
| Pseudo R-squared | 0.0162 |

Standard Errors in parentheses

*** p<0.01, ** p<0.05, * p<0.1

Table 33: Self-Reported Understanding of the problems that the LGBTQ community is facing in the country.

|  | Baseline: |
| --- | --- |
|  | Placebo Cue |
|  |  |
| Traditional Cue | 1.188 |
|  | (0.662) |
| Political Interest | 1.098* |
|  | (0.054) |
| Traditional Cue*Political Interest | 0.962 |
|  | (0.084) |
| Political Cue | 1.156 |
|  | (0.361) |
| Religious Cue | 0.849 |
|  | (0.270) |
| Age | 0.941 |
|  | (0.105) |
| Gender | 1.099 |
|  | (0.249) |
| Ethnicity | 0.991 |
|  | (0.020) |
| Political Interest | 1.015 |
|  | (0.016) |
| Constant | 0.088*** |
|  | (0.051) |
|  |  |
| Observations | 676 |
| Pseudo R-squared | 0.00988 |

Standard Errors in parentheses

*** p<0.01, ** p<0.05, * p<0.1

Table 34: Self-Reported Understanding of the problems that the LGBTQ community is facing in the country.

|  | Baseline: |
| --- | --- |
|  | Traditional Cue |
|  |  |
| Placebo Cue | 0.674 |
|  | (0.402) |
| Political Interest | 1.065 |
|  | (0.052) |
| Placebo Cue*Political Interest | 1.082 |
|  | (0.099) |
| Political Cue | 1.201 |
|  | (0.374) |
| Religious Cue | 0.884 |
|  | (0.281) |
| Age | 0.945 |
|  | (0.106) |
| Gender | 1.091 |
|  | (0.247) |
| Ethnicity | 0.990 |
|  | (0.020) |
| Political Party | 1.015 |
|  | (0.016) |
| Constant | 0.102*** |
|  | (0.060) |
|  |  |
| Observations | 676 |
| Pseudo R-squared | 0.0109 |

Standard Errors in parentheses

*** p<0.01, ** p<0.05, * p<0.1

Table 35: Self-Reported Understanding of the problems that the LGBTQ community is facing in the country.

|  | Baseline: |
| --- | --- |
|  | Political Cue |
|  |  |
| Placebo Cue | 0.863 |
|  | (0.269) |
| Traditional Cue | 0.835 |
|  | (0.260) |
| Religious Cue | 0.620 |
|  | (0.376) |
| Political Interest | 1.079 |
|  | (0.052) |
| Religious Cue*Political Interest | 1.031 |
|  | (0.094) |
| Age | 0.942 |
|  | (0.105) |
| Gender | 1.105 |
|  | (0.250) |
| Ethnicity | 0.991 |
|  | (0.020) |
| Political Party | 1.014 |
|  | (0.016) |
| Constant | 0.113*** |
|  | (0.067) |
|  |  |
| Observations | 676 |
| Pseudo R-squared | 0.00973 |

Standard Errors in parentheses

*** p<0.01, ** p<0.05, * p<0.1

Table 36: Self-Reported Understanding of the problems that the LGBTQ community is facing in the country.

|  | Baseline: |
| --- | --- |
|  | Religious Cue |
|  |  |
| Placebo Cue | 1.180 |
|  | (0.376) |
| Traditional Cue | 1.139 |
|  | (0.363) |
| Political Cue | 1.935 |
|  | (1.110) |
| Political Interest | 1.104** |
|  | (0.053) |
| Political Cue*Political Interest | 0.937 |
|  | (0.084) |
| Age | 0.945 |
|  | (0.106) |
| Gender | 1.104 |
|  | (0.250) |
| Ethnicity | 0.990 |
|  | (0.020) |
| Political Party | 1.014 |
|  | (0.016) |
| Constant | 0.074*** |
|  | (0.043) |
|  |  |
| Observations | 676 |
| Pseudo R-squared | 0.0105 |

Standard Errors in parentheses

*** p<0.01, ** p<0.05, * p<0.1

# Balance Checks

Table 37: Randomization Checks

|  | (1) | (2) | (3) | (4) |
| --- | --- | --- | --- | --- |
|  | Placebo Cue | Traditional Cue | Political Cue | Religious Cue |
|  |  |  |  |  |
| Age | 0.960 | 0.994 | 1.092 | 0.963 |
|  | (0.079) | (0.081) | (0.090) | (0.079) |
| Gender | 0.941 | 1.074 | 1.064 | 0.935 |
|  | (0.155) | (0.179) | (0.182) | (0.154) |
| Region | 0.996 | 0.991 | 0.996 | 1.017 |
|  | (0.020) | (0.020) | (0.021) | (0.021) |
| Ethnicity | 1.014 | 0.980 | 1.004 | 1.002 |
|  | (0.016) | (0.016) | (0.016) | (0.016) |
| Political Party | 0.996 | 1.009 | 1.003 | 0.993 |
|  | (0.011) | (0.012) | (0.012) | (0.011) |
| Political Interest | 1.001 | 0.987 | 0.984 | 1.028 |
|  | (0.031) | (0.031) | (0.032) | (0.032) |
| Constant | 0.407** | 0.366** | 0.237*** | 0.340*** |
|  | (0.166) | (0.151) | (0.101) | (0.138) |
|  |  |  |  |  |
| Observations | 791 | 791 | 791 | 791 |
| Pseudo R-squared | 0.00139 | 0.00453 | 0.00168 | 0.00310 |

Standard Errors in parentheses

*** p<0.01, ** p<0.05, * p<0.1

# Survey Instrument

**Demographic questions**

Q Residential Status (enumerator answers the question)

- Rural
- Urban

Q Region (enumerator answers)

- 1 Western Region (WR)
- 2 Western North (WN)
- 3 Central Region (CR)
- 4 Greater Accra Region (GAR)
- 5 Volta Region (VR)
- 6 Oti Region
- 7 Eastern Region (ER)
- 8 Ashanti Region (AR)
- 9 Bono Region
- 10 Bono East Region
- 11 Ahafo Region
- 12 Northern Region (NR)
- 13 Savana Region
- 14 North East Region
- 15 Upper East Region (UER)
- 16 Upper West Region (UWR)

Q How old are you?

- 18-29
- 30-39
- 40-49
- 50-59
- 60+

Q What is your gender?

- Male
- Female
- Other

Q Which ethnic groups do you belong to? Mark the space or spaces which apply to you.

- Akan
- Mole-Dagbon
- Ewe
- Ga-Adangbe
- Konkomba
- Guan
- Gurunsi
- Kusasi
- Bissa
- Other

Q Do you approve of the way President Nana Akufo-Addo is handling his job as the president of Ghana?  1 = not at all, 2 = somewhat, 3 = moderately, 4 =very much, 5= very strongly.

- Not at all approve
- Somewhat approve
- Moderately approve
- Very Much approve
- Very Strongly approve

Q Among the political parties listed here, which party, if any, do you feel closest to?

- New Patriotic Party (NPP)
- National Democratic Congress (NDC)
- Convention People's Party (CPP)
- Peoples National Convention (PNC)
- Progressive People’s Party (PPP)
- National Democratic Party (NDP)
- Other
- Not Affiliated with any party

Q How much do you trust each of the following institutions?

|  | 0 - Not at all | 1 - Just a little | 2 -Somewhat | 3 - A lot |
| --- | --- | --- | --- | --- |
| The President |  |  |  |  |
| Parliament |  |  |  |  |
| Metropolitan/Municipal/District Chief Executive |  |  |  |  |
| Police |  |  |  |  |
| Ghana Armed Forces |  |  |  |  |
| Ministry of Foreign Affairs |  |  |  |  |
| The Judiciary |  |  |  |  |

Q Overall, how satisfied or dissatisfied are you with the way democracy works in Ghana?

- 1 very dissatisfied;
- 2 somewhat dissatisfied;
- 3 neutral;
- 4 somewhat satisfied;
- 5 very satisfied

Q Here is a list of actions that people sometimes take as citizens when they are dissatisfied with government performance. For each of these, please tell me whether you, personally, have done any of these things during the past year. If not, would you do this if you had the chance: Participated in a demonstration or protest march.

- No, would never do this,
- No, but would do if had the chance,
- Yes, once or twice,
- Yes, several times,
- Yes, often,
- Don’t know,
- Refused to answer

Q Please indicate whether you have been involved in any of the listed activities.

|  | Never | One Time | Sometimes | Several Times | On a regular basis | Do not Know | Refused to answer |
| --- | --- | --- | --- | --- | --- | --- | --- |
| Joined others in your community to request action from government |  |  |  |  |  |  |  |
| Contacted a government official |  |  |  |  |  |  |  |
| Participated in a demonstration or protest march |  |  |  |  |  |  |  |
| Voting |  |  |  |  |  |  |  |
| How closely do you follow national politics? |  |  |  |  |  |  |  |

Q Please tell me whether you would like having people from this group as neighbors, dislike it, or not care: Homosexuals?

- Strongly Dislike
- Somewhat Dislike
- Neither Dislike or Like
- Somewhat Like
- Strongly Like

Q There are many ways to govern a country. Would you disapprove or approve of the following alternatives: Only one political party is allowed to stand for election and hold office.

- Strongly Disapprove
- Disapprove
- Neither approve nor disapprove
- Approve
- Strongly Approve

Q There are many ways to govern a country. Would you disapprove or approve of the following alternatives: The army comes in to govern the country

- Strongly Disapprove
- Disapprove
- Neither approve nor disapprove
- Approve
- Strongly Approve

Q There are many ways to govern a country. Would you disapprove or approve of the following alternatives: Elections and Parliament are abolished so that the president can decide everything?

- Strongly Disapprove
- Disapprove
- Neither approve nor disapprove
- Approve
- Strongly Approve

**[Each respondent will be randomly assigned to one of the treatment conditions below.]**

Treatment 1 Random person speaking on an unrelated subject.

*“Some ordinary Ghanaians want to encourage all of us to lead a healthy lifestyle. All Ghanaians should consider their health in their life choices.*

*All of us should take our bodies seriously and have regular medical checks with our doctors while keeping a healthy diet.*

*We should find ways to discuss our health problems and find ways to combat them, thus improving the resilience of our communities.”*

Treatment 2 Traditional Leaders

*“In the face of recent controversies about LGBTQI+ issues Traditional Leaders [enumerator - mention the name of the paramount chief] in your area have made the following remarks: We in Ghana respect each other and live peacefully. We do not encourage or support hate against any member of the community. You are aware that there have been issues of LGBTQI+ in our community in recent times.*

*Our community is ruled by laws, and members of the LGTB community are part of our society, and like all of us our laws protect them from any form of discrimination and harm. They, like us, contribute to our society through paying taxes and participating in community service; they take care of their families.*

*As a result, I appeal to all members of the community to treat members of the LGBTQI+ community with respect and accord them rights they deserve under our laws. “*

Treatment 3

*“In the face of recent controversies about LGTBQI+ issues leaders of the [Party matched to the Respondent's preselected party affiliation] including the MP from this area have made the following remarks.*

*In Ghana among our neighbors and abroad, we are known to be tolerant and peaceful people and are a people who respect the fundamental human rights of all persons. I believe you are all aware of the current issues in the country about the human rights of the LGBTQI+ community.*

*We are all Ghanaians, and as a country, we believe that we must be tolerant of all persons in our society as long as they do not break any laws.*

*The fundamental law of our land is the constitution, and the constitution grants fundamental human rights to everyone, including members of the LGBTQI+ community. I, therefore, appeal to all Ghanaians to respect the fundamental rights of the LGBTQI+ community.”*

Treatment 4

“*In the face of recent controversies about LGBTQ+ issues the leader of your [enumerator to mention the name of the church/mosque] has said that followers of Christ/Allah, love all persons just like God loves us. Just like Christ/Allah accepts all persons with open arms because we are his children, we accept all persons with open arms and show kindness and mercy to all. In that same vein, we appeal to all children of God to show kindness and mercy to all persons, including the LGBTQI+ community.*

*For us Christians/Muslims, the Bible/Koran teaches us to show love and kindness to our fellow humans and reserve judgment for God. For this reason, we would love all Christians/Muslims to treat all LGBTQI+ persons with love and kindness.“*

**Post-treatment questions [Presented in randomized order]**

Q Will you support or oppose LGBTQQI+ rights in Ghana?

 Please select your answer using 1-11 scale, 1 indicates Strongly Oppose while 11 indicates Strongly Support.  

|  | 1 - Strongly Oppose | 2 | 3 | 4 | 5 | 6 - Neither Oppose nor Support | 7 | 8 | 9 | 10 | 11 - Strongly Support |
| --- | --- | --- | --- | --- | --- | --- | --- | --- | --- | --- | --- |
| Select your answer |  |  |  |  |  |  |  |  |  |  |  |

Q To what extent you agree with the statement: I understand the difficulties that members of the LGBTQQI+ community in Ghana are experiencing

 Please select your answer using 1-11 scale, 1 indicates Strongly Oppose while 11 indicates Strongly Support.

|  | 1 - Strongly Oppose | 2 | 3 | 4 | 5 | 6 - Neither Oppose nor Support | 7 | 8 | 9 | 10 | 11 - Strongly Support |
| --- | --- | --- | --- | --- | --- | --- | --- | --- | --- | --- | --- |
| Select your answer |  |  |  |  |  |  |  |  |  |  |  |

Q To what extent you agree with the statement: I am willing to do business with members of the LGBTQQI+ community in Ghana

 Please select your answer using 1-11 scale, 1 indicates Strongly Oppose while 11 indicates Strongly Support.

|  | 1 - Strongly Oppose | 2 | 3 | 4 | 5 | 6 - Neither Oppose nor Support | 7 | 8 | 9 | 10 | 11 - Strongly Support |
| --- | --- | --- | --- | --- | --- | --- | --- | --- | --- | --- | --- |
| Select your answer |  |  |  |  |  |  |  |  |  |  |  |

Q To what extent you agree with the statement: I will be comfortable living in a residential community with members of the LGBTQQI+ community in Ghana

 Please select your answer using 1-11 scale, 1 indicates Strongly Oppose while 11 indicates Strongly Support.

|  | 1 - Strongly Oppose | 2 | 3 | 4 | 5 | 6 - Neither Oppose nor Support | 7 | 8 | 9 | 10 | 11 - Strongly Support |
| --- | --- | --- | --- | --- | --- | --- | --- | --- | --- | --- | --- |
| Select your answer |  |  |  |  |  |  |  |  |  |  |  |

Q To what extent you agree with the statement: Members of the LGBTQQ in Ghana should be able to participate in community meetings.

 Please select your answer using 1-11 scale, 1 indicates Strongly Oppose while 11 indicates Strongly Support.

|  | 1 - Strongly Oppose | 2 | 3 | 4 | 5 | 6 - Neither Oppose nor Support | 7 | 8 | 9 | 10 | 11 - Strongly Support |
| --- | --- | --- | --- | --- | --- | --- | --- | --- | --- | --- | --- |
| Select your answer |  |  |  |  |  |  |  |  |  |  |  |

Q How likely is it that you are going to vote for political candidates who are pro-LGBTQQ

 Please select your answer using 1-11 scale, 1 indicates Strongly Oppose while 11 indicates Strongly Support.

|  | 1 - Strongly Oppose | 2 | 3 | 4 | 5 | 6 - Neither oppose nor Support | 7 | 8 | 9 | 10 | 11 - Strongly Support |
| --- | --- | --- | --- | --- | --- | --- | --- | --- | --- | --- | --- |
| Select your answer |  |  |  |  |  |  |  |  |  |  |  |

Q To what extent you support government policies that restrict media information about LGBTQQ communities in Ghana 
  
 Please select your answer using 1-11 scale, 1 indicates Strongly Oppose while 11 indicates Strongly Support.

|  | 1 - Strongly Oppose | 2 | 3 | 4 | 5 | 6 - Neither Oppose nor Support | 7 | 8 | 9 | 10 | 11 - Strongly Support |
| --- | --- | --- | --- | --- | --- | --- | --- | --- | --- | --- | --- |
| Select your answer |  |  |  |  |  |  |  |  |  |  |  |

Q Did the text mention the issue of rights for the LGBTQQI+ communities in Ghana?

- Yes
- No

# References

Ghana Statistical Service (2000) Ghana Living Standard Survey, Accra: Ghana Statistical Service

# Inclusivity in global research

**Ethical considerations, permits and authorship.**

Provide details as to who granted permissions and/or consent for the study to take place in the Methods section of your manuscript. This should include the names of **all** ethics boards, governmental organizations, community leaders or other bodies that provided approval for the study. If individuals provided approval refer to these people by their role or title but do not list their name(s).

Reported on page 18 of the Manuscript. This study was approved by the IRB board at North Carolina State University’s led by the coordinator of administration. The study was given an exempt.

If there were any deviations from the study protocol after approval was obtained, please provide details of these changes in the Methods section of your manuscript.

N/A

Did this study involve local collaborators that are residents of the country where the research was conducted, or members of the community studied? If you do not have any authors from said communities, please provide an explanation for this below.

| The study involved 32 local field researchers/enumerators. Each of the local field researchers possessing detailed local knowledge about the cultural norms administrated the survey according to pre-determined sampling procedures explained on pages 18 in the Manuscript. |
| --- |

Everyone listed as an author should meet PLOS’ criteria for authorship and all individuals who meet these criteria should be included in the author byline, rather than the acknowledgements. For further information please see the journal’s Authorship Policy.

**Human subjects research (e.g. health research, medical research, cross-cultural psychology)**

Did you obtain written informed consent from a representative of the local community or region before the research took place? How did you establish who speaks for the community? Details of written informed consent obtained from study participants should be reported separately in the Methods section of your manuscript.

All participants were asked to provide their verbal and written consent before participating in the study. See pages 18 in the Manuscript.

How did members of the local community provide input on the aims of the research investigation, its methodology, and its anticipated outcome(s)?

When engaging with the local community, how did you ensure that the informed consent documents and other materials could be understood by local stakeholders?

Field researchers came from every sampled region in Ghana. Each of the local field researchers was indigenous for the region with detailed knowledge of the local population and previous experience with similar studies. In addition, the local field researchers provided detailed consent forms in both written and verbal format from the participants and the explained the procedure of the experiment, including the nature of the questions. After their response has been finalized the local field researchers debriefed each of the participants and explained to them in verbal and written form the purpose of the study. To the best of our knowledge the study did not generate threats to our participants, enumerators, and supervisors.

Will the findings of the research be made available in an understandable format to stakeholders in the community where the study was conducted (e.g. via a presentation, summary report, copies of publications, etc.)? Please provide details of how this will be achieved.

N/A

**Non-human subjects research using specimens/ animals collected as part of the study, or those housed in archival collections. Examples include archaeology, paleontology, botany and zoology.**

Did the permission you obtained from a local authority to perform the study include an agreement on access to outputs and benefit sharing? This may include procedures to enable fair distribution of the benefits and resources arising from the research performed. Please include any details of Prior Informed Consent and Benefit Sharing Agreements obtained. These may be required by field-specific regulations, for example the Convention on Biological Diversity (CBD) and the associated Nagoya Protocol.

N/A

If the material used in your study was imported, please A) provide the year it was imported and B) indicate whether permits were obtained to import/export the materials used, C) provide details of any permits obtained. If this information is not available, please indicate this.

N/A

If you used archival specimens, please state how the material used in your study was acquired by the institute it is held in and provide details of any permits obtained for the original excavations/ sample collection. If this information is not available, please indicate this.

N/A

How was the potential cultural significance of the materials collected in your study to local communities considered in your research design? Were Indigenous peoples and/or local researchers and institutions involved with archaeological excavations / collection of specimens? If so, please provide a description of their involvement.

N/A

If your manuscript includes photographs of human remains please indicate whether authors obtained permission from descendants or affiliated cultural communities to do so.

N/A
